# Violated Expectations in the Cyberball Paradigm: Testing the Expectancy Account of Social Participation With ERP

**DOI:** 10.3389/fpsyg.2018.01762

**Published:** 2018-09-25

**Authors:** Katharina Schuck, Michael Niedeggen, Rudolf Kerschreiter

**Affiliations:** ^1^Division of Experimental Psychology and Neuropsychology, Department of Education and Psychology, Free University of Berlin, Berlin, Germany; ^2^Division of Social, Organizational, and Economic Psychology, Department of Education and Psychology, Free University of Berlin, Berlin, Germany

**Keywords:** cyberball, expectancy, probability, verticality, ERP, experimental design

## Abstract

Previous social exclusion experiments identified two factors affecting the participants’ evaluation of participation in a virtual ball tossing game (cyberball): ball reception probability and vertical position of the participant’s avatar on the screen. The P3 component in the event-related brain potentials (ERPs) indicated that both factors moderate subjective expectancies on social participation. The present research builds on an expectancy model explaining these effects and tests whether its predictions – established in a within-participant design – also hold in a between-participant design more common in behavioral cyberball studies. Participants were randomly assigned to four conditions which differed in ball reception probability (16% vs. 26%) and the avatar’s vertical position (inferior vs. superior). To track the state of expectancy of involvement online, we recorded the ERP response evoked by ball receptions of the participant. Retrospectively, social involvement and social need threat were rated in a questionnaire. As hypothesized, low ball reception probability elicited enlarged P3 amplitudes in the ERPs, increased negative mood, and threatened social needs. For participants at inferior position, ERP and questionnaire effects were less expressed. This effect of verticality can be traced back to an adjustment in the expected involvement as signaled by a differential adaptation of the P3 amplitude within an experimental run. These results confirm that the predictions of an expectancy model also apply to cyberball studies using a between-participant design. However, the comparison with the results of previous within-participant design studies suggests that the sensitivity of the adjustment processes critically depends on the choice of the experimental design.

## Introduction

**FIGURE 1:**
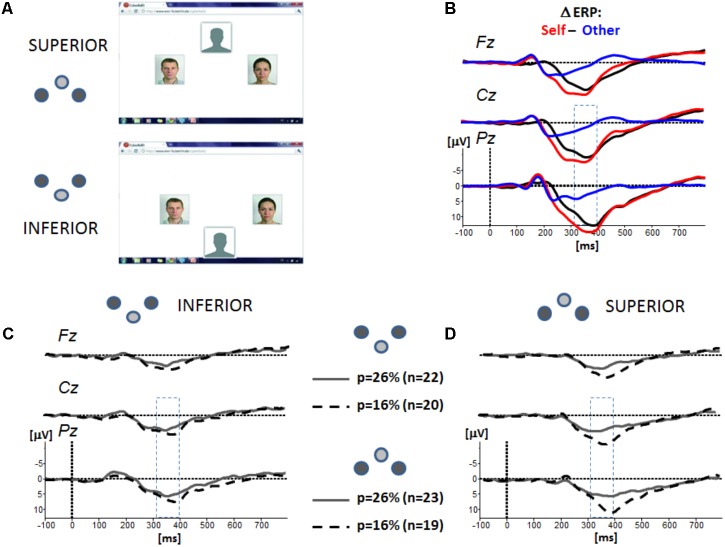
**(A)** Display for the experimental groups (inferior vs. superior), and the corresponding icons used thereafter. The position of the two putative co-players was centered horizontally, and, depending on group assignment, the position of the participant’s avatar varied vertically. The photos representing co-players were not real photos, but morphs. Presentation of the ball in spatial proximity to the participants’ avatar signaled “ball possession” and requested the participant to move the ball to a co-player by pressing a corresponding button on a keyboard. Co-players’ ball possession randomly lasted between 400–1400 ms. After the participants key press or a varying co-player ball possession time, the ball vanished for 500 ms before reappearing in proximity to another avatar. **(B)** Analysis of ERP effects was based on the difference waves computed for the conditions “ball reception of the participants (self)” (red trace) vs. “ball reception of the co-players (others)” (blue trace). **(C,D)** Grand-averaged difference ERPs (negativity up) separated for the experimental groups at inferior **(C)** and superior **(D)** position. Superimposed are the traces for the experimental groups with low exclusion (*p* = 26%) and high exclusion (*p* = 16%). At the four midline electrodes (AFz, Fz, Cz, and Pz), the P3 amplitude is mostly expressed at centro-parietal positions in the time range from 340 to 420 ms.

Being neglected in social interaction is an aversive experience. It directly impacts our affective state (Twenge et al., 2001) and mental health (Macdonald and Leary, 2005). To examine the psychological consequences of social exclusion, Williams et al. (2000) introduced the cyberball paradigm which simulates exclusion in a virtual ball tossing game (see **Figure 1A**). The participant is represented as an avatar on a computer screen, and two other avatars putatively represent human co-players connected via internet. The co-players, however, are computer-generated, and pass the ball back and forth in so-called “exclusionary" rallies. Retrospectively, the participants’ reports indicate that not receiving the ball anymore or even just receiving the ball less frequently threatens fundamental social needs (belonging, self-esteem, control, and meaningful existence), and elicits negative mood (Zadro et al., 2004; Williams, 2007). A recent meta-analysis (Hartgerink et al., 2015), based on 120 cyberball studies, confirmed the reliability of the exclusionary effect on self-reports, and estimated a large effect size (*d* > 1.4). As a consequence of the reliable exclusion effects and their large effect size that could be retrieved with this easy to implement paradigm, cyberball emerged as the gold standard in experimental research on the effects of social exclusion. Correspondingly, the paradigm has been used beyond the field of Social Psychology, for instance in Clinical Psychology (Renneberg et al., 2012; Engel et al., 2016; Fung and Alden, 2017), Developmental Psychology (Pharo et al., 2011; Will et al., 2013; Wolfer and Scheithauer, 2013), or Health Psychology (Stock et al., 2013; Pieritz et al., 2017).

To further elucidate the cognitive processes involved in the processing of social exclusion, Weschke and Niedeggen (2013, 2015), Niedeggen et al. (2017), and Weschke and Niedeggen (2016) additionally recorded event-related brain potentials (ERPs). Specifically, participants first ran an inclusionary block (probability of ball reception: 33%), immediately followed by a partial-exclusion block (probability of ball reception: 16%). As shown in previous work, this setup induces the exclusionary effects reliably. Accordingly, in self-reports significant effects on the threat of social needs and negative mood. The ERP results indicated that a transition from inclusion to partial exclusion was associated with an increase of a centro-parietal positivity at about 350 ms (Gutz et al., 2011; Weschke and Niedeggen, 2013). This ERP component evoked by the task-relevant event (ball reception) resembles the characteristics of the P3 (Polich, 2007). Comparable to the effect of subjective probability in an *oddball paradigm* (Duncan-Johnson and Donchin, 1977), the P3 amplitude reflects the degree of expectancy violation triggered by the transition from inclusion to partial exclusion in cyberball (Weschke and Niedeggen, 2015). In other words, the P3 amplitude reflects the deviation from an expected event, here defined by the previous experience of social involvement.

The violation of expectancy, however, critically depends on the psychological factor verticality that is built into the experimental set up of the cyberball paradigm because the avatar of the participant is presented *below* the avatars of the two co-players on the computer screen. As verticality is known to affect a wide range of psychological states, such as goals, motives, and emotions (Hall et al., 2005), it is likely to also impact the processing of social exclusion. Most consistently, an effect of verticality on the self-attribution of social power has been found (Schubert, 2005): Whereas a “high” position indicates control over others, a “low” position signals a subordinate status (Fiske, 1992). From a social embodiment perspective, verticality is not a mere metaphor, but activates goals and motives automatically, and serves as a perceptual symbol of power (Giessner and Schubert, 2007). Consequently, our social interactions are inevitably affected by information on the vertical status.

Since the standardized experimental setup of the cyberball paradigm includes verticality, the activation of the psychological mechanism attached to verticality can be assumed to also affect the processing of social exclusion in the cyberball game. First evidence for this idea was provided by Schoel et al. (2014) based on a *between-participant design*. They argued that the vertical position of the participant in the standardized setup below both co-players might serve as a perceptual symbol of social power. Schoel et al. (2014) flipped the standard setup and found a significant modulation of the exclusionary effects: In the group of participants, in which the avatar of the participant was positioned above the putative co-players, the threat of social needs was less expressed than in the group of participants with an avatar at an inferior position. The impact of verticality on the participants’ evaluation supports the idea that the feeling of need threat induced by ostracism can be modulated by self-assigned social power.

Niedeggen et al. (2017) also hypothesized that verticality affects the self-assignment of social power but questioned the reduced sensitivity of the superior position. The exclusionary effect was induced in a *within-participant design* by a transition from an inclusionary block (ball reception 33%) to partial-exclusion (ball reception: 16%). As predicted, the effect of exclusion in self-reports depended on the vertical position of the participants: compared to superior and even vertical position – regarding the position of the putative co-players – the increase in need threat and negative mood were less expressed for participants at inferior position. In contrast to the findings of Schoel et al. (2014), this lower spatial position prepared the participants for being neglected by the co-players. As revealed by the analysis of ERPs, this process is associated with a shift in subjective expectancies: At an inferior position, the effect on the P3 amplitude (Δ[inclusion – exclusion]) is significantly reduced as compared to even or superior position. Hence, the vertical position affects the processing of the exclusionary event: Participants with an avatar at an inferior position appeared to be prepared for exclusion, so that the deviance to the *a priori* expected involvement was less expressed.

The parallel pattern of behavioral and electrophysiological results in the Niedeggen et al. (2017) study strongly support the conclusion that self-assigned power as influenced by vertical position biases the subjective expectation of social participation. According to the model of social exclusion by Kerr and Levine (2008), the deviation between expected and perceived social participation determines the evaluation of social participation. Moreover, the degree of deviation reflects an inconsistency within the cognitive system which is assumed to trigger aversive arousal (Proulx et al., 2012). At an inferior position, the bias in expectancy reduces the inconsistency, and exclusionary events are therefore experienced as less aversive.

The differences in outcome between the studies of Schoel et al. (2014) and Niedeggen et al. (2017) direct our attention to two crucial factors in the experimental design and setup of cyberball studies: First, most cyberball studies are based on between-participant designs which imply that participants receive either an inclusionary or an exclusionary condition. In contrast, in cyberball studies which are based on within-participant designs, participants receive an inclusionary rally followed by an exclusionary rally. Apart from obviously having a larger statistical power, a *within*-design allows to modify the participants expectation of involvement. As a participants’ evaluation of exclusion can be modified by the previous experience of an inclusionary rally (Gutz et al., 2011), effects in self reports and ERPs might be differently pronounced. Second, the majority of behavioral (i.e., non-neuroscience) cyberball studies are restricted in length: the number of throws rarely exceeds 30 throws (Hartgerink et al., 2015). In contrast, cyberball studies applying ERPs require a higher number of experimental trials (100 throws or more) which bears the possibility of changes over time within one rally. Investigating these changes can yield additional insight into the processing of participation. In an earlier ERP study Kawamoto et al. (2013) observed a marked reduction of the P3 amplitude over time. Following an expectancy account, such a process might signal an adaptation process which should affect the self-report on the experience of social participation.

With the present study, we aim to test whether the predictions of an expectancy account on social exclusion also apply for the commonly used between-participant design. We ask whether the behavioral and ERP correlates of social exclusion observed previously (Niedeggen et al., 2017) can also be observed if a within-participant baseline is not provided. Moreover, we ask whether the assumed bias in expectancy on participation induced by verticality also applies to the between-participant design. Finally, we take advantage of the longer runs necessary for ERP studies in order to explore systematic changes of the ERP signal over time.

The predictions derived from the expectancy account on social exclusion will be detailed in the following:

First, an expectancy account predicts that the ERP signature signaling expectancy violation in the cyberball paradigm does not necessarily require a preceding inclusionary experience and can also be found in a between-participant design. Our main argument is that an exclusionary event *per se* violates participants’ *a priori* expectation of participation. Specifically, we predicted that the violation of the *a priori* expectation affects the expression of the P3 amplitude: Following our previous results, the P3 amplitude is expected to be more pronounced in a high as compared to a low exclusion condition (Hypothesis 1a). A corresponding effect is expected for the subjective evaluation of social participation (Hypothesis 1b).

Second, an expectancy account predicts that a biasing process induced by vertical position also applies to a *between*-design. A previous within-participant study (Niedeggen et al., 2017) indicated that the vertical position of the participants’ avatar provides a bias in the expectancy of involvement, and therefore affects the sensitivity for a transition from an inclusionary to an exclusionary condition. This process should also apply to between-participant designs: Building on the same argument as above, the vertical position should *per se* modulate the *a priori* expectation of involvement in the game. In other words, the bias on expectancy induced by verticality does not necessarily require an immediately preceding inclusionary condition. Hence, we predicted that ERP effects (Hypothesis 2a) and self-reports (Hypothesis 2b) signal a reduced expectation of participation and a corresponding reduced need-threat for participants with avatars located at inferior as compared to superior position.

Third, following an expectancy account, systematic changes in the P3 amplitude in the time course of inclusionary and/or exclusionary runs can be related to a recalibration process. In line with previous findings (Kawamoto et al., 2013), we hypothesized that the P3 amplitude will be reduced over time (Hypothesis 3a). The biasing effect of vertical position [see above, (Niedeggen et al., 2017)] might also affect the recalibration of expectation: We assume that an adaptation of the *a priori* expectation of participation is more strongly expressed in participants with avatars located at inferior as compared to superior position (Hypothesis 3b).

## Materials and Methods

### Participants

The local ethics committee approved the experimental procedure. All participants provided their written consent for participating according to the Declaration of Helsinki. Based on previous results on the effect of verticality (Niedeggen et al., 2017), mid to high-sized effects of the experimental factors (probability, vertical position) were expected for the questionnaire and the ERP data. According to a G^∗^Power (Erdfelder et al., 1996) power analysis, a total sample of 90 participants would be needed to detect medium-sized to strong effects [$\eta_{p}^{2}$ = 0.30, adjusted to the taxonomy of (Cohen, 1988)] with a power of 80% using a *F* test with alpha at 0.05. With the final sample size of *N* = 84, a power of 0.78 to detect the expected effects was achieved.

In total, 97 participants were examined. We excluded thirteen participants due to a too low number of single EEG sweeps (less than 20) available in at least one experimental condition following a strict artifact rejection (criteria: see below). The number of remaining sweeps was insufficient to provide a reliable averaged ERP signal following the split-half analysis of the EEG data. The remaining 84 participants (age *M* = 22.3 years, *SD* = 4.38 years, range: 18–36 years, 55 female, 29 male) had self-reportedly no history of psychiatric or neurological disorders. Participants were randomly assigned to one of the four experimental conditions resulting from orthogonally combining vertical position (inferior vs. superior) with two reduced probabilities of ball reception: (26% vs. 16% of all ball throws.

### Task and Design

The experimental setup (programmed in MATLAB; R2012a, The MathWorks, Inc.) consisted of the cyberball game, which was preceded by a cover story task about training visual imagery. Details on the experimental task have already been described elsewhere (Niedeggen et al., 2017). In order to allow a comparison of effects, the setup of the study – including instruction, cover story, visual presentation, and timing of events – followed the setup of a previous within-participant design experiment (Niedeggen et al., 2017).

All participants were told that they took part in a study testing visual imagination capabilities, and a corresponding short questionnaire about visual imagination ability (Vividness of Visual Imagery Questionnaire, Marks, 1973) was to be completed as a supposed training of visual imagery. The setup of the cyberball game is depicted in **Figure 1A**: Participants were told that they would play a ball-tossing game with two other co-players connected via internet. The computer display (7° × 7° at a viewing distance of 120 cm) featured the photos of two putatively connected co-players. The photos of the co-players were presented continuously, whereas the presentation of the ball was dynamic. Presentation of the ball in spatial proximity to the participants’ avatar signaled “ball possession.” By pressing a keyboard button, the participant could select the player to whom she/he wanted to throw the ball. No speeded response of the participant was required and a systematic effect of experimental factors on response time was not found (see **Supplementary Data Sheet 4**). Participants were instructed to restrict eye movements to the area on the screen defined by ball positions (approximately 2° × 2°). Co-players’ ball possession lasted randomly between 400 and 1.400 ms. After the participants key press or a varying player ball possession time, the ball vanished for 500 ms before reappearing in proximity to another avatar. To adapt to the technical requirements of ERP recording and to reduce eye movements, the ball position was indicated as a stationary cue, and a ball trajectory was not displayed.

The position of the photos of the two putatively connected co-players was centered with respect to verticality. The position of the avatar of the participants’ avatar was centered horizontally, but its vertical position depended on the group assignment. In the condition “inferior” and “superior,” the avatar was positioned 2.6° below or above of the co-players, respectively. The distance of the participants’ avatar to the photos of the co-players was comparable (3°) in all conditions.

Following the instructions and a short training introduction (20 ball throws), the participants played one block of the cyberball game. Depending on random assignment, participants either received the ball with a slightly reduced probability (26% of all ball throws, *lowEXC*, low partial exclusion), or with a highly reduced probability (16% of all ball throws, *highEXC*, high partial exclusion). The probabilities and their classification (low vs. high partial exclusion) refer to a “fair” ball reception expectation of 33% (given the number of co-players). The total number of ball throws was 250 in the 16%-condition (*highEXC*) and 200 in the 26%-condition (*lowEXC*). Correspondingly, the number of a ball receptions of the participant was 40 in the 16%-condition and 52 in the 26%-condition.

Immediately following the ball tossing game, participants were asked to estimate the frequency of ball reception (manipulation check), and to fill out the Need Threat Questionnaire (NTQ). The NTQ measures the effect of exclusion on the perceived level of social need threat (scales: belonging, self-esteem, meaningful existence, and control) and on negative mood (Williams et al., 2000). Each scale consists of three items to be rated on 5-point Likert scales (ranging from 1 “not at all” to 5 “completely”), assessing how much each item applies to the subject, the mood index is calculated from eight different items. Its validity has been confirmed in numerous studies based on the cyberball paradigm (Hartgerink et al., 2015). As there is evidence that the credibility of the cover story does neither affect the rating of the social need threat (Zadro et al., 2004), nor the expression of the P3 component (Weschke and Niedeggen, 2013), the questionnaire did not include an item to check credibility. Moreover, previous studies did not indicate that verticality affects the credibility of the cover story (Niedeggen et al., 2017). After completing all the questionnaires, participants were fully debriefed and gave informed consent again.

### EEG Recording

Six active Ag/AgCl electrodes were positioned at midline (AFz, Fz, Cz, and Pz) and lateral fronto-central positions (FC5, FC6). FCz served as the ground electrode, Active electrodes (impedance <5 kΩ) were referenced to linked earlobes. Using EEG-8 amplifiers (Contact Precision Instruments, Cambridge, United Kingdom), biosignals were recorded continuously at a sampling rate of 500 Hz, and an online bandpass filter (0.01–100 Hz). To control for ocular artifacts, vertical and horizontal electrooculogram (EOG) were recorded. Off-line, EEG data were analyzed using “Brain Vision Analyzer” (Version 1.05, Brain Products GmbH, Gilching, Germany). EEG data were offline-filtered (0.3–30 Hz, 24 dB/Oct) and segmented with respect to the onset of ball reception of the participant (“self”) or one of the co-players (“others”). Please note, that the latter event did not consider trials in which the participant was the sender. Epoch length of each segment extended from −100 to 700 ms. Each segment was corrected to baseline (−100 to 0 ms).

EEG trials with muscular or ocular artifacts (e.g., blinks) were excluded from analysis. In a first run, artifacts were detected semi-automatically by applying an amplitude criterion (>80 μV). In a subsequent visual inspection, trials were eliminated if this criterion (a) applies to the horizontal or vertical EOG, (b) applies to the baseline period of an EEG segment, and (c) signals sustained alpha activity in the EEG. Furthermore, trials were controlled for slow drifts (linear deviations from baseline extending for more than 300 ms) and high-frequency bursts (electric activity exceeding 50 Hz, and 40 μV).

Following this rigorous procedure, 42% of the trials (*n* = 250) were rejected in the *highEXC* condition, leaving a mean number of artifact-free trials of 145.48 (*SD* = 22.08). This includes a mean number of trials of 29.0 (*SD* = 5.05) for the event “self” and 116.48 (*SD* = 19.65) for the event “other.” In the *lowEXC* condition, artifacts were more frequent and 48% of the trials (*n* = 200) were rejected, leaving a mean number of artifact-free trials of 103.38 (*SD* = 15.54). This includes a mean number of trials of 32.24 (*SD* = 5.13) for the event “self” and 71.13 (*SD* = 10.94) for the event “other.” Due to the differences in rejection rate, the mean number of trials for the event “self” included in the averaging procedure did not differ significantly between the 16%- and the 26%-condition, *F*(1,82) = 3,226, *p* = 0.076, $\eta_{p}^{2}$ = 0.038. Due to the partial exclusion of the participants across all conditions, there were more segments for the event “ball reception of the co-player” (other) as compared to the event “ball reception of the participant” (self). Therefore, the number of EEG segments in the conditions “other” was adjusted to the number of segments in the “self” condition by random selection in each participant.

### Data Analysis

#### EEG Data

Within a participant, ERPs were separately averaged for the factor “ball recipient” (self vs. other), and electrode position. Between subjects, the factors “position” (inferior vs. superior) and “probability” (*lowEXC* vs. *highEXC*) were considered. For each participant, the difference ERP for the conditions “self” – “other” was computed. This procedure has already been used in a previous study (Gutz et al., 2011), and accounts for interindividual differences in ERP amplitudes which could mask experimental effects in between-participant designs. Our analysis of the data also showed that ERPs to the event “other” (ball reception of the co-players) were not significantly modulated by the experimental factors “position” or “probability” (**Supplementary Data Sheet 1**). Experimental effects are therefore due to systematic effect on the ERP response to ball reception of the participant (“self”).

Following the inspection of the grand-averaged difference ERP (self – others), the maximum of the P3 was found at centro-parietal positions at about 380 ms. Considering that the P3 has a sustained time course, the mean amplitude was estimated for each participant in the time range extending from 340 to 420 ms. The analysis was restricted to a time window of 80 ms in order to allow a comparison with the results of a previous experiment (Niedeggen et al., 2017). Exported mean amplitudes in this time range were analyzed using SPSS (version 22, IBM). To analyze effects of the experimental manipulations on the P3 amplitude (self – others), an ANOVA was calculated including the between-participant factors “position” and “probability,” and the within-participant factor “electrode position.” Analysis was restricted to the centro-parietal midline electrodes (Cz, Pz). As in previous studies (Gutz et al., 2011; Niedeggen et al., 2014; Weschke and Niedeggen, 2015), P3 amplitudes were most expressed at these sites. The results of the ANOVA are reported with Greenhouse-Geisser corrected degrees of freedom and *p*-values, if indicated. In case of significant interactions of the experimental factors, corresponding *post hoc* comparisons were performed.

To analyze systematic changes within an experimental block of the P3 amplitude, we performed a split-half analysis: ERP data of each participant were separately averaged for the first and second half of the artifact-free set of preprocessed sweeps (factor “half”: 1st vs. 2nd). The averaged ERP was at least based on ten sweeps, and visual inspection ensured that the ERP component of interest could be identified reliably in the condition “self.” For each half, difference waves were computed (ball reception “self” – “others”), and mean amplitudes for the time range 340–420 ms were computed. The extracted data were analyzed running an ANOVA including the within factors “half” (1st vs. 2nd) and electrode position (Cz, Pz), and the between factors “verticality” (inferior vs. superior) and “probability” (partial exclusion “low” vs. “high”). *Post hoc* test was performed in case of an interaction with the factor “half.” The split-half analysis is a rather conservative approach to analyze the variability of the P3. This technique has already been used in a previous cyberball study (Kawamoto et al., 2013), confirming that systematic changes in amplitude can be detected.

The differences in vertical position of the avatar also induced different ocular activity which became apparent in the vEOG signal. However, eye movements are unlikely to contribute to the effect of verticality on the P3 described below: Significant differences in the vEOG signal depending on the vertical position of the participant’s avatar were expressed in the time range of interest [340–420 ms: *F*(1,80) = 67.73, *p* < 0.001, $\eta_{p}^{2}$ = 0.458], but also in the preceding time range [240–320 ms: *F*(1,80) = 63.09, *p* < 0.001, $\eta_{p}^{2}$ = 0.438]. ERP effects of verticality, however, were only observed in the P3 range (340–420 ms, see below), but not in the preceding time range (see **Supplementary Data Sheet 6**). Moreover, the effect of verticality on the P3 was restricted to the second half of the experimental block (see below), whereas the vEOG effect of verticality was observed in both halves (see **Supplementary Data Sheets 4, 5**).

#### Questionnaire Data

Participants’ questionnaire data (estimated frequency of ball reception, four NTQ scales, negative mood) were analyzed using SPSS (version 22, IBM). To check reliability of NTQ measures for the present sample, Cronbach’s alpha was calculated for each scale, ensuring high internal consistency for scales “self,” “existence,” and negative mood (α = 0.824–892). Internal consistency was lower for scales “belonging” (α = 0.642) and “control” (α = 0.583). Please note that homogeneity of variances (Levene test) was asserted for all but one scale: For the scale “meaningful existence,” equal variances between groups could not be assumed (*p* < 0.001). For this reason, the results of the appropriate Brown-Forsythe-Test will be reported for this scale additionally, in the result section.

The data were separately analyzed running 2 × 2 ANOVAs, including the between-participants factors “verticality” and “probability.” In a first run, the mean NTQ score summarized over the four scales was analyzed. As the four NTQ scales (belonging, self-esteem, meaningful existence, and control) refer to different psychological constructs (Williams and Nida, 2011) and are correspondingly affected selectively by experimental manipulation (Schoel et al., 2014), they were additionally analyzed independently. Reported degrees of freedom and *p*-values were corrected according to Greenhouse-Geisser, and as for the ERP data, corrected *p*-values will be reported. In case of significant interactions, *post hoc* comparisons were performed.

## Results

Descriptive statistics on the questionnaire data and P3 amplitudes are summarized in **Table 1** (Raw data: see **Supplementary Data Sheets 2, 3**).

**Table 1:** Means for the questionnaire and ERP data, separated for group assignment (position: inferior vs. superior) and ball reception probability (*p* = 26% [*lowEXC*] vs. *p* = 16% [*highEXC*]).

Position	Inferior		Superior	
Probability	*p* = 26%	*p* = 16%	*p* = 26%	*p* = 16%
Estimated ball reception [%]	26.0 [1.9] [22.3, 29.7]	13.4 [2.0] [9.5, 17.2]	25.2 [1.8] [21.6, 28.8]	14.3 [2.0] [10.3, 18.2]
NTQ mean	3,49 [0.13] [3.22, 3.75]	2,94 [0.14] [2.67, 3.22]	3.25 [0.13] [2.99, 3.51]	2.49 [0.14] [2.21, 2.77]
NTQ: belonging	3.56 [0.19] [3.18, 3.94]	3.03 [0.20] [2.64, 3.43]	3.48 [0.19] [3.11, 3.85]	2.67 [0.21] [2.26, 3.07]
NTQ: self-esteem	3.50 [0.17] [3.15, 3.85]	3.13 [0.18] [2.77, 3.50]	3.22 [0.17] [2.88, 3.56]	2.49 [0.19] [2.12, 2.87]
NTQ: meaningful existence	4.59 [0.21] [4.17, 5.01]	3.45 [0.22] [3.01, 3.89]	4.06 [0.21] [3.65, 4.47]	3.11 [0.23] [2.66, 3.56]
NTQ: control	2.29 [0.22] [1.85, 2.72]	2.15 [0.23] [1.70, 2.61]	2.24 [0.21] [1.81, 2.66]	1.70 [0.24] [1.23, 2.17]
Negative mood	8.34 [0.61] [7.12, 9.56]	8.90 [0.64] [7.62, 10.18]	9.48 [0.60] [8.28, 10.67]	11.34 [0.66] [10.03, 12.66]
P3 amplitude	4.92 [0.83] [3.27, 6.57]	6.81 [0.87] [5.08, 8.55]	5.21 [0.81] [3.59, 6.82]	10.37 [0.89] [8.59, 12.14]
P3 amplitude (1st half)	6.29 [0.76] [4.75, 7.83]	7.55 [0.80] [5.93, 9.17]	5.92 [1.05] [3.81, 8.05]	11.87 [1.18] [9.47, 14.26]
P3 amplitude (2nd half)	5.17 [0.72] [3.71, 6.63]	6.48 [0.76] [4.95, 8.02]	5.96 [0.93] [4.08, 7.83]	12.77 [1.05] [10.65, 14.87]

*First row refers to the mean and standard error of mean, the second row to the upper and lower limits of confidence intervals (95%).*

### Estimated Frequency of Ball Perception

**FIGURE 2:**
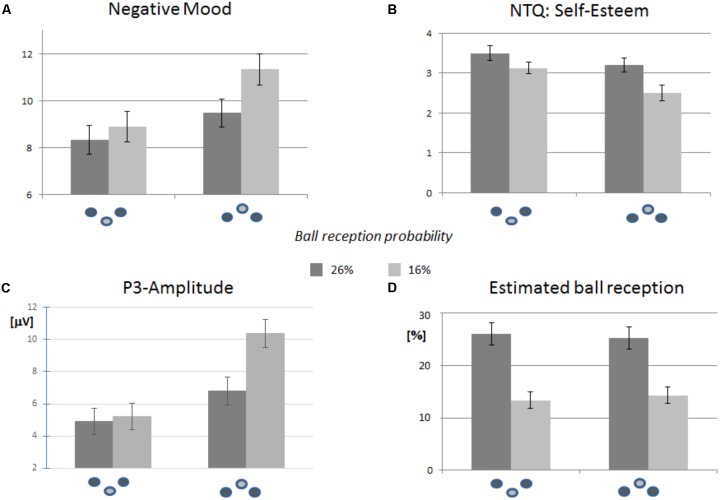
Descriptive statistics for the self-report and ERP data, separated for the experimental factors “position” (inferior vs. superior) and “probability” (16% vs. 26%). Error bars refer to the standard error of the mean. The icons refer to the vertical position of the participant (inferior vs. superior). **(A)** Negative mood was more pronounced in participants at superior position, and increased with by higher exclusion probability. **(B)** The same pattern was observed for the NTQ scale “self-esteem.” **(C)** Decreasing the probability of ball reception had an increasing effect on P3 amplitude. The increase was more pronounced in participants at superior position. **(D)** The estimated ball reception confirmed that participants noticed the probability of ball reception in all four experimental groups correctly.

The retrospective estimation of the frequency of ball reception served as a manipulation check and ensures that the variation of the degree of exclusion was perceived. This manipulation check (see **Figure 2D**) confirmed that both conditions were perceived reliably: The factor “probability” discriminated between the experimental groups and that the percentage of ball reception was correctly estimated. The factor “verticality” did not influence the participants’ estimation. The ANOVA confirmed the main effect of “probability,” *F*(1,80) = 38.231, *p* < 0.001, $\eta_{p}^{2}$ = 0.323, but did not reveal a main effect of “verticality,” or an interaction.

### NTQ Scales

The mean NTQ score summarizing the four scales signaled that social needs were threatened to a stronger degree in the *highEXC* as compared to the *lowEXC* condition, *F*(1,80) = 22.923, *p* < 0.001, $\eta_{p}^{2}$ = 0.223. The level of social need threat was significantly more expressed in participants with avatars at a superior position, *F*(1,80) = 6.410, *p* = 0.013, $\eta_{p}^{2}$ = 0.074. An interaction of the factors was not detected.

For all single NTQ scales and across positions, satisfaction of social needs was reduced to a stronger degree in the *highEXC* condition than in the *lowEXC* condition (see **Table 1**). This difference yielded significant main effects in the analyses of factor “probability” for the scales “belonging,” *F*(1,80) = 11.639, *p* = 0.001, $\eta_{p}^{2}$ = 0.128, “self-esteem,” *F*(1,80) = 9.323, *p* = 0.003, $\eta_{p}^{2}$ = 0.104, and “meaningful existence,” *F*(1,80) = 23.533, *p* < 0.001, $\eta_{p}^{2}$ = 0.227, but did not reach significance for “control.” The need threat appears to be more expressed for the groups at superior position, independently of the degree of partial exclusion. Corresponding differences reached significance for the scales “self-esteem,” *F*(1,80) = 6.623, *p* = 0.012, $\eta_{p}^{2}$ = 0.076 (see also: **Figure 2B**), and “meaningful existence,” *F*(1,80) = 4.149, *p* = 0.045, $\eta_{p}^{2}$ = 0.049. No interaction between factors was detected for any of the NTQ scales.

The additional Brown-Forsythe-Test indicated for the scale “meaningful existence” (see section “Materials and Methods”) confirmed a significant effect of the factor “probability,” *F*(1,80) = 21.941, *p* < 0.001, but showed only a marginal effect of the factor “position,” *F*(1,80) = 3.031, *p* = 0.085.

### Negative Mood

Negative mood (**Figure 2A**) was enhanced by decreasing probability of ball reception, although the ANOVA barely failed to show a significant difference, *F*(1,80) = 3.696, *p* = 0.058, $\eta_{p}^{2}$ = 0.044. The effect of verticality was more clearly expressed at superior position, indicating a more negative mood, *F*(1,80) = 38.067, *p* = 0.006, $\eta_{p}^{2}$ = 0.092. Although an effect of probability appeared to be more expressed at superior position, no significant interaction was found.

### P3 Amplitude

The grand-averaged ERPs rely on differences waves (ball reception “self” – “other”). As depicted in **Figure 1B**, the grand-averaged ERP for the event “ball reception other” is characterized by a phasic negativity at 180 ms, and a phasic positivity at 220 ms returning to baseline. The negativity was also expressed for the event “ball reception self,” whereas the positivity was more expressed and sustained. The difference wave was primarily dominated by this positive shift which was pronounced at centro-parietal sites with a maximum at about 380 ms. As mentioned above, the statistical analysis was based on the mean amplitude in the time range 340–420 ms and considered the midline electrodes Cz and Pz. Please note that the ERP response to the event “others” was not affected by the experimental factors “verticality” and “probability.” The corresponding analysis of the amplitude effects is provided in the **Supplementary Data Sheet 1**.

Independent of vertical group assignment, the P3 amplitude was increased in the condition *highEXC* as compared to *lowEXC* (**Figures 1C,D**, **2C**). This pattern was confirmed by a significant effect in the ANOVA, *F*(1,80) = 17.166, *p* < 0.001, $\eta_{p}^{2}$ = 0.177. Overall, the mean P3 amplitudes were also increased in participants in superior positions. Again, the ANOVA confirmed the main effect of “verticality,” *F*(1,80) = 5.075, *p* = 0.027, $\eta_{p}^{2}$ = 0.060. As for negative mood, the effect of “probability” appears to be more strongly expressed in the superior as compared to the inferior group. The ANOVA, however, barely failed to confirm a significant effect, *F*(1,80) = 3.694, *p* = 0.058, $\eta_{p}^{2}$ = 0.044.

### Split-Half Effect of the P3 Amplitude

**FIGURE 3:**
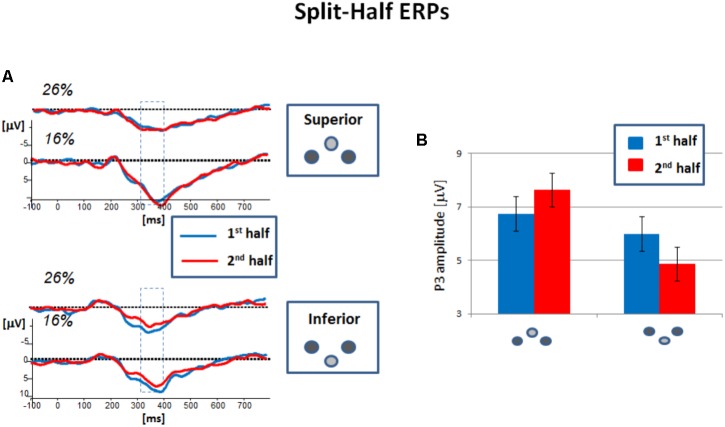
Grand-averaged ERP responses to the event “ball reception” in the cyberball game. ERP responses in the first and second half of an experimental run are superimposed and separated for the probability conditions (26% ball reception, 16% ball reception). **(A)** For participants with an avatar at superior position, the amplitudes – here depicted for electrode Pz – are slightly increased. This effect was obtained for both probability conditions. **(B)** For participants with an avatar at inferior position, Pz amplitudes are clearly reduced and this effect was obtained for both probability conditions. **(C)** Mean amplitudes in the P3 range compared for the first and second half. The reduction in the inferior group was found to be significant. Error bars refer to the standard error of the mean.

To analyze systematic variations of the P3 within a block of the cyberball game, we conducted a split-half analysis of the ERP data. To this end, the ERP responses in the first and in the second half of the cyberball game were compared. The corresponding grand-averaged ERPs (difference waves self-other) are depicted in **Figure 3A** for the parietal electrode Pz.

In the temporal window of interest, the P3 amplitude appeared to remain stable for the participants at superior position. In contrast, the amplitudes were decreased in the second half for participants at inferior position. **Figure 3B** indicates that the amplitude reduction is comparably expressed for both levels of the factor “probability,” *lowEXC* and *highEXC*.

The ANOVA confirmed the impression that the effect of the factor “half” was differently expressed in the experimental groups and depended on verticality (see **Figure 3C**). The analysis did not reveal a significant main effect of the factor “half,” but a significant interaction with the factor “verticality,” *F*(1, 80) = 4.899, *p* = 0.030, $\eta_{p}^{2}$ = 0.058. Both were not found to be modulated by the factor “probability.”

The follow-up comparisons indicated no significant differences between first and second half in the superior group [*F*(1,39) = 0.749, *p* = 0.392, $\eta_{p}^{2}$ = 0.019]. However, they showed a significant reduction of P3 amplitude for the inferior groups in the second half of the block, *F*(1,40) = 5.752, *p* = 0.021, $\eta_{p}^{2}$ = 0.126).

## Discussion

The present research tested the prediction of an expectancy violation account on the processing of social exclusion in a between-participant design. In addition to self-reports, ERPs provided an online measurement of the participants’ evaluation processes. We predicted that an exclusionary event *per se* violates participants’ *a priori* expectation of participation. Accordingly, this violation should affect the expression of the P3 amplitude (Hypothesis 1a), as well as the subjective evaluation of social participation (Hypothesis 1b). Furthermore, we predicted ERPs (Hypothesis 2a) and self-reports (Hypothesis 2b) to signal a reduced expectation of participation for participants with avatars located at inferior position. Finally, we hypothesized that the P3 amplitude will be reduced over time (Hypothesis 3a) and that the adaptation of the *a priori* expectation of participation is more strongly expressed at inferior as compared to superior position (Hypothesis 3b). To test these predictions, we orthogonally combined vertical position of the participants’ avatar with respect to the co-players (inferior vs. superior) with two reduced probabilities of ball reception in a between-participants design.

The results of the ERP and questionnaire data confirmed our hypotheses. First, we observed the ERP signature of an expectancy violation in exclusionary blocks (Hypothesis 1a), and the corresponding effects on self-reports (Hypothesis 1b). Second, vertical position affected the ERP signature of exclusion (Hypothesis 2a) and the self-reports (Hypothesis 2b) indicating a reduced expectation of participation for players with avatars at inferior position. Third, the level of the P3 amplitude decreased within an experimental block (Hypothesis 3a). As expected, this effect was closely linked to the vertical position of a participant and can be related to a selective recalibration of expectancy for participants with avatars located at inferior position (Hypothesis 3b). Taken together, this pattern of results consistently supports the notion that prediction of an expectancy account can be confirmed using a between-participant designs. In the following, we provide a more thorough analysis of the three main findings.

### Processing of Exclusionary Events in the Between-Participant Design

The effect of partial exclusion on the P3 effect is in line with previous studies (Gutz et al., 2011; Weschke and Niedeggen, 2013) reporting a significant increase of the P3 amplitude with transition to a low probability of ball reception. Furthermore, recent research provided evidence that this ERP effect depends on the violation of expectation rather than on probability: If probability for ball reception is reduced, but not unexpected (i.e., by increasing the number of co-players), the P3 amplitude is not increased and the feeling of exclusion is not reported in the self-reports (Weschke and Niedeggen, 2015).

However, an unexpected reduction of the probability of ball reception leads to an increase in P3 amplitude, and correspondingly, the self-reported threat to social needs (NTQ) and aversive affective state is expressed to a larger degree. The P3 amplitude therefore serves as the neurophysiological manifestation of an expectancy violation process, which can be related to a neural network underlying the computation of the probabilistic structure of relevant events (Strange et al., 2005; Mars et al., 2008). With this study, we support the empirical evidence for an expectancy violation process in the evaluation of social participation that has already been stressed in theoretical models (Lepoire and Burgoon, 1994; Kerr and Levine, 2008). Moreover, the fact that we could extend the ERP effect to the between-participant design in the present research confirms that the expectancy violation account does not necessarily require an immediately preceding inclusionary experience. This supports the crucial role of *a priori* expectations of participation that has already been highlighted in clinical studies on individuals with Borderline personality disorder (Gutz et al., 2015).

### The Verticality Effect in the Between-Participant Design

Following previous results (Niedeggen et al., 2017), we hypothesized that participants at inferior position are biased toward exclusionary events. The present research confirmed that the effect of verticality extends to the between-participant design for both, P3 effects and self-reports: The reduced P3 amplitude effects suggested that partial exclusion was less surprising at an inferior position. Accordingly, aversive affect was less expressed, and threats to social needs (here: self-esteem and meaningful existence) were reduced. Note that this “preparedness for exclusion” elicited by verticality did not affect the estimated percentage of ball perception (see **Figure 2D**).

A possible candidate for the mechanism mediating the main effect of verticality on sensitivity to social exclusion could be a differential self-assignment of social power (Schubert, 2005). An inferior position signals that participants are less powerful which biases the processing of exclusionary events. A corresponding mechanism has been described for self-relevant injustice (Sawaoka et al., 2015).

Note that the replication of the verticality effect running a *between*-design supports the idea that vertical position biases the *a priori* expectation of involvement in the game, and not exclusively the sensitivity for a transition from inclusion to exclusion. This conclusion will be discussed in more detail below.

### Verticality-Specific Adaptation Process of the P3 Amplitude

The split-half analysis of the P3 data confirmed that fluctuations in amplitude may signal systematic changes of the cognitive state (Mars et al., 2008). First evidence for such a psychophysiological process within the cyberball game was provided by Kawamoto et al. (2013): Within a block of total exclusion, the P3 amplitude triggered by the event “ball reception of the co-player” was significantly reduced when comparing the first and the second half of the block. According to the authors, the effect is due to a shift in the distribution of attentional resources: More attention is supposed to be directed to exclusionary cues in the initial stages of an interaction. When participants realize that they never receive the ball, attention is moved away from these cues.

Our data confirm a reduction of the P3 amplitude within a block, but the effect was observed for the relevant event “*self*,” i.e., the ball reception of the participant. This result does not support the idea of an attentional shift because our split half effect can be observed in both, high and low partial exclusion conditions. Following Kawamoto’s line of reasoning, in neither of these conditions a re-direction of attention should be obtainable since occasional ball reception was always provided. More importantly, the ERP split-half effect is restricted to participants with an avatar at inferior position.

As expected, the reduction of the P3 amplitude within a block of the cyberball game is therefore in line with an adjustment process in the participants’ expectation of participation. The adjustment process, in turn, affects the ratings the retrospective self-reports. These processes can be modulated by vertical position associated with social power: An inferior position signals low social power, and the participant re-adjusts the level of expected participation more rapidly to recurrent aversive exclusionary events. Due to this adaptation process, retrospective self-reports signal a reduced need threat. In contrast, a superior position signals an enhanced social power. As signaled by the P3, participants do not adjust the level of expectancy to recurrent aversive events. Correspondingly, social need threat is expressed highly in the retrospective self-reports.

Consider that the standard cyberball procedure is restricted to 30–40 ball throws (Hartgerink et al., 2015), and will therefore fail to detect differential adaptation effects depending on vertical position. This difference in the experimental setup is also likely to contribute to the findings of Schoel et al. (2014) indicating a higher sensitivity in participants with avatars at inferior position.

Please note that the differential reduction of the P3 amplitude is also speaks to the question whether the effect of verticality might be due to differences in the sensory processing of stimuli in the upper and lower visual field. For early visually evoked potentials, differences in latency and amplitude have been reported (Lee et al., 2009; Hagler, 2014) reflecting differences in the underlying neuroanatomy. In our data, however, the effect of verticality was not significantly pronounced in the first half (superior vs. inferior: *F*(1,81) = 2.426, *p* = 0.123, $\eta_{p}^{2}$ = 0.029), but only in the second half (superior vs. inferior: *F*(1,81) = 9.702, *p* = 0.003, $\eta_{p}^{2}$ = 0.107) of the experiment block. If differences in early sensory processing trigger the P3 amplitude effect, it should have been expressed in both halves of the experiment. Moreover, visual field asymmetries were not observed in a passive (and non-social) oddball task with a visual target event defined either in the upper or in the lower visual field: Data of this pilot study (*n* = 14 participants) did not reveal differences in the P3 amplitude depending on the vertical position of the target (see **Supplementary Data Sheet 7**). Together, this pattern of results supports our idea that the effect of verticality on the P3 amplitude is reliable beyond differences in the sensory processing of stimuli in the upper and lower visual field.

### Integration of the Results of Between- and Within-Participant Designs

As mentioned before, the experimental setup – including the experimental factors “probability” and “verticality” – has also been used in a previous experiment running a within-participant design (Niedeggen et al., 2017). This enables a tentative comparison of the confidence intervals and effect sizes to evaluate whether the choice of the experimental design affects the sensitivity of the ERP and questionnaire effects.

With respect to the experimental factor “probability of ball reception” the sensitivity of the ERP markers seems to be reduced in a between-participants design. In the present study, the mean of the “probability” effect (conditions *highEXC* – *lowEXC*) was 6.62 μV (95% CI: 2.92–10.31) indicating a moderate effect size ($\eta_{p}^{2}$ = 0.15). In the previous within-participant study, the corresponding effect was clearly more pronounced with a mean “probability” effect of 11.64 μV (95% CI: 9.29–13.98) indicating a large effect size [$\eta_{p}^{2}$ = 0.73, (Niedeggen et al., 2017)]. Please note that both studies did not differ with regard to probability of ball reception (partial exclusion, 16%). Moreover, we did not observe ERP differences for the event “ball perception of co-players” (others) in this study, whereas previous studies – relying on a within-participant design (Kawamoto et al., 2013; Themanson et al., 2013; Weschke and Niedeggen, 2015) – reported such a P3 effect of exclusion.

The self-report data confirm the differences in sensitivity for exclusion depending on the choice of the experimental design: Combining the four NTQ scales, we observed a mean “probability” effect of 0.641 (95% CI: 0.364–0.919) indicating a moderate effect size ($\eta_{p}^{2}$ = 0.205) in the between-participant design of the present study. In contrast, when running a *within-*design the mean “probability” effect was as twice as large (*M* = 1.216, 95% CI: 0.930–1.503), and indicated a large effect size ($\eta_{p}^{2}$ = 0.642) (Niedeggen et al., 2017).

The differences in sensitivity seems to extend to the second experimental factor, “vertical position”: In a previous within-participant study (Niedeggen et al., 2017) effects of verticality (inferior vs. superior) were significantly increased by a transition from an inclusionary to an exclusionary condition. This interaction of verticality and exclusionary status ($\eta_{p}^{2}$ = 0.21) signaled that the verticality does not affect the processing of an *a priori* expectation of involvement (fair condition). Rather, it biases the processing of the transition from expected (inclusion) to unexpected (exclusion) social participation. Although the present results tend to replicate this interaction with respect to the P3 effects (see **Figure 2C**, $\eta_{p}^{2}$ = 0.04), the interaction is not replicable for self-reports: Here, the vertical position apparently biases negative affect and threat to social needs independently of the degree of partial exclusion.

In sum, we observed a noticeable reduction in sensitivity to the experimental factors “probability” and “verticality” when running a between-participant design. ERP effect sizes were degraded and the interaction of experimental factors (namely, verticality and degree of exclusion) seemed to rely on the previous experience of inclusion. We assume that a preceding inclusionary run provided the participant with a more-reliable within-participant baseline and served the adjustment of expectation. The assumed process of adjustment is consistent with theoretical models of social exclusion (Lepoire and Burgoon, 1994; Kerr and Levine, 2008), and can be integrated in a more-general model on inconsistency compensation (Proulx et al., 2012).

## Conclusion

The present research confirms that the predictions of an expectancy account on social participation can be extended to the between-participant design typically used in behavioral cyberball studies. Put differently, exclusionary events *per se* violate participants’ *a priori* expectation of participation. Moreover, we identified ERP markers for a temporal adaptation of expectancy of involvement which depends on the vertical position: Whereas subjective expectancy for participation remained stable at a superior position, we find a gradually reduced subjective expectation of participation at an inferior position during virtual interaction in the cyberball game. In other words, the “preparedness for exclusion” associated with the inferior position(Niedeggen et al., 2017) becomes more apparent in long- rather than in short-lasting interactions. Consequently, group differences relying on the re-adjustment of expectation processes will not become visible if interactions are restricted in time.

Integration of the present research with previous studies suggests that the experimental design affects the processing of social exclusion in the cyberball paradigm. The reduced effects sizes in the between-participant designs could be due to a lack of a reliable within-participant baseline of expectation. A promising avenue for future research would therefore be to directly test possible mechanisms responsible for the reduced effects sizes found in the between-participant design.

## Ethics Statement

EthicsCommittee of the Department of Education and Psychology, FU Berlin. Approval no.: 106/2015 (Verarbeitung von sozialem Ausschluss im Cyberball-Paradigma: Kognitions- und sozialpsychologische Moderatoren). The local ethics committee approved the experimental procedure. All participants provided their written consent for participating according to the Declaration of Helsinki. All participants received a cover story (study intended to test visual imagination capabilities). After completing the experiment, all participants were fully debriefed and gave informed consent again. The procedure is detailed in the manuscript.

## Author Contributions

All authors designed the study, contributed to the data analysis (questionnaires, ERP), interpreted the results, and prepared and commented the manuscript. KS prepared the experiment and collected the data.

## Conflict of Interest Statement

The authors declare that the research was conducted in the absence of any commercial or financial relationships that could be construed as a potential conflict of interest.

## References

[B1] Cohen J. (1988). *Statistical Power Analysis for the Behavioral Sciences.* Hillsdale, NJ: L. Erlbaum Associates.

[B2] Duncan-Johnson C. C. Donchin E. (1977). On quantifying surprise: the variation of event-related potentials with subjective probability. *Psychophysiology* 14 456–467. 10.1111/j.1469-8986.1977.tb01312.x 905483

[B3] Engel M. Fritzsche A. Lincoln T. M. (2016). Anticipation and experience of emotions in patients with schizophrenia and negative symptoms. An experimental study in a social context. *Schizophr. Res.* 170 191–197. 10.1016/j.schres.2015.11.028 26673972

[B4] Erdfelder E. Faul F. Buchner A. (1996). Gpower: a general power analysis program. *Behav. Res. Methods Instrum. Comput.* 28 1–11. 10.3758/BF03203630

[B5] Fiske A. P. (1992). The 4 elementary forms of sociality - framework for a unified theory of social-relations. *Psychol. Rev.* 99 689–723. 10.1037/0033-295X.99.4.689 1454904

[B6] Fung K. Alden L. E. (2017). Once hurt, twice shy: social pain contributes to social anxiety. *Emotion* 17 231–239. 10.1037/emo0000223 27606825

[B7] Giessner S. R. Schubert T. W. (2007). High in the hierarchy: how vertical location and judgments of leaders’ power are interrelated. *Organ. Behav. Hum. Decis. Process.* 104 30–44. 10.1016/j.obhdp.2006.10.001

[B8] Gutz L. Kuepper C. Renneberg B. Niedeggen M. (2011). Processing social participation: an event-related brain potential study. *Neuroreport* 22 453–458. 10.1097/WNR.0b013e3283476b67 21558970

[B9] Gutz L. Renneberg B. Roepke S. Niedeggen M. (2015). Neural processing of social participation in borderline personality disorder and social anxiety disorder. *J. Abnorm. Psychol.* 124 421–431. 10.1037/a0038614 25603358

[B10] Hagler D. J. Jr (2014). Visual field asymmetries in visual evoked responses. *J. Vis.* 14:13. 10.1167/14.14.13 25527151 PMC4274841

[B11] Hall J. A. Coats E. J. Lebeau L. S. (2005). Nonverbal behavior and the vertical dimension of social relations: a meta-analysis. *Psychol. Bull.* 131 898–924. 10.1037/0033-2909.131.6.898 16351328

[B12] Hartgerink C. H. J. Van Beest I. Wicherts J. M. Williams K. D. (2015). The ordinal effects of ostracism: a meta-analysis of 120 cyberball studies. *PLoS One* 10:e0127002. 10.1371/journal.pone.0127002 26023925 PMC4449005

[B13] Kawamoto T. Nittono H. Ura M. (2013). Cognitive, affective, and motivational changes during ostracism: an ERP, EMG, and EEG study using a computerized cyberball task. *Neurosci. J.* 2013:304674. 10.1155/2013/304674 26317090 PMC4437265

[B14] Kerr N. L. Levine J. M. (2008). The detection of social exclusion: evolution and beyond. *Group Dyn. Theor. Res. Pract.* 12 39–52. 10.1111/mec.13803 27540705 PMC5054864

[B15] Lee B. Kaneoke Y. Kakigi R. Sakai Y. (2009). Human brain response to visual stimulus between lower/upper visual fields and cerebral hemispheres. *Int. J. Psychophysiol.* 74 81–87. 10.1016/j.ijpsycho.2009.07.005 19643151

[B16] Lepoire B. A. Burgoon J. K. (1994). 2 contrasting explanations of involvement violations - expectancy violations theory versus discrepancy arousal theory. *Hum. Commun. Res.* 20 560–591.

[B17] Macdonald G. Leary M. R. (2005). Why does social exclusion hurt? The relationship between social and physical pain. *Psychol. Bull.* 131 202–223. 10.1037/0033-2909.131.2.202 15740417

[B18] Marks D. F. (1973). Visual imagery differences in recall of pictures. *Br. J. Psychol.* 64 17–24. 10.1111/j.2044-8295.1973.tb01322.x 4742442

[B19] Mars R. B. Debener S. Gladwin T. E. Harrison L. M. Haggard P. Rothwell J. C. (2008). Trial-by-trial fluctuations in the event-related electroencephalogram reflect dynamic changes in the degree of surprise. *J. Neurosci.* 28 12539–12545. 10.1523/JNEUROSCI.2925-08.2008 19020046 PMC6671727

[B20] Niedeggen M. Kerschreiter R. Hirte D. Weschke S. (2017). Being low prepares for being neglected: verticality affects expectancy of social participation. *Psychon. Bull. Rev.* 24 574–581. 10.3758/s13423-016-1115-5 27368640

[B21] Niedeggen M. Sarauli N. Cacciola S. Weschke S. (2014). Are there benefits of social overinclusion? Behavioral and ERP effects in the Cyberball paradigm. *Front. Hum. Neurosci.* 8:935. 10.3389/fnhum.2014.00935 25477807 PMC4237054

[B22] Pharo H. Gross J. Richardson R. Hayne H. (2011). Age-related changes in the effect of ostracism. *Soc. Influ.* 6 22–38. 10.1080/15534510.2010.525852

[B23] Pieritz K. Schafer S. J. Strahler J. Rief W. Euteneuer F. (2017). Chronic stress moderates the impact of social exclusion on pain tolerance: an experimental investigation. *J. Pain Res.* 10 1155–1162. 10.2147/JPR.S129872 28553136 PMC5440009

[B24] Polich J. (2007). Updating P300: an integrative theory of P3a and P3b. *Clin. Neurophysiol.* 118 2128–2148. 10.1016/j.clinph.2007.04.019 17573239 PMC2715154

[B25] Proulx T. Inzlicht M. Harmon-Jones E. (2012). Understanding all inconsistency compensation as a palliative response to violated expectations. *Trends Cogn. Sci.* 16 285–291. 10.1016/j.tics.2012.04.002 22516239

[B26] Renneberg B. Herm K. Hahn A. Staebler K. Lammers C. H. Roepke S. (2012). Perception of social participation in borderline personality disorder. *Clin. Psychol. Psychother.* 19 473–480. 10.1002/cpp.772 22076727

[B27] Sawaoka T. Hughes B. L. Ambady N. (2015). Power heightens sensitivity to unfairness against the self. *Pers. Soc. Psychol. Bull.* 41 1023–1035. 10.1177/0146167215588755 26048859

[B28] Schoel C. Eck J. Greifeneder R. (2014). A matter of vertical position: consequences of ostracism differ for those above versus below its perpetrators. *Soc. Psychol. Personal. Sci.* 5 149–157. 10.1177/1948550613488953

[B29] Schubert T. W. (2005). Your highness: vertical positions as perceptual symbols of power. *J. Pers. Soc. Psychol.* 89 1–21. 10.1037/0022-3514.89.1.1 16060739

[B30] Stock M. L. Gibbons F. X. Peterson L. M. Gerrard M. (2013). The effects of racial discrimination on the hiv-risk cognitions and behaviors of black adolescents and young adults. *Health Psychol.* 32 543–550. 10.1037/a0028815 23646837 PMC3649873

[B31] Strange B. A. Duggins A. Penny W. Dolan R. J. Friston K. J. (2005). Information theory, novelty and hippocampal responses: unpredicted or unpredictable? *Neural. Netw.* 18 225–230. 10.1016/j.neunet.2004.12.004 15896570

[B32] Themanson J. R. Khatcherian S. M. Ball A. B. Rosen P. J. (2013). An event-related examination of neural activity during social interactions. *Soc. Cogn. Affect. Neurosci.* 8 727–733. 10.1093/scan/nss058 22577169 PMC3739919

[B33] Twenge J. M. Baumeister R. F. Tice D. M. Stucke T. S. (2001). If you can’t join them, beat them: effects of social exclusion on aggressive behavior. *J. Pers. Soc. Psychol.* 81 1058–1069. 10.1037/0022-3514.81.6.1058 11761307

[B34] Weschke S. Niedeggen M. (2013). The effect of the physical presence of co-players on perceived ostracism and event-related brain potentials in the cyberball paradigm. *PLoS One* 8:e71928. 10.1371/journal.pone.0071928 23951269 PMC3739783

[B35] Weschke S. Niedeggen M. (2015). ERP effects and perceived exclusion in the Cyberball paradigm: correlates of expectancy violation? *Brain Res.* 1624 265–274. 10.1016/j.brainres.2015.07.038 26236023

[B36] Weschke S. Niedeggen M. (2016). Target and non-target processing during oddball and cyberball: a comparative event-related potential study. *PLoS One* 11:e0153941. 10.1371/journal.pone.0153941 27100787 PMC4839683

[B37] Will G. J. Van Den Bos W. Crone E. A. Guroglu B. (2013). Acting on observed social exclusion: developmental perspectives on punishment of excluders and compensation of victims. *Dev. Psychol.* 49 2236–2244. 10.1037/a0032299 23544860

[B38] Williams K. D. (2007). Ostracism. *Annu. Rev. Psychol.* 58 425–452. 10.1146/annurev.psych.58.110405.085641 16968209

[B39] Williams K. D. Cheung C. K. Choi W. (2000). Cyberostracism: effects of being ignored over the internet. *J. Pers. Soc. Psychol.* 79 748–762. 10.1037/0022-3514.79.5.748 11079239

[B40] Williams K. D. Nida S. A. (2011). Ostracism: consequences and coping. *Curr. Dir. Psychol. Sci.* 20 71–75. 10.1177/0963721411402480 16968209

[B41] Wolfer R. Scheithauer H. (2013). Ostracism in childhood and adolescence: emotional, cognitive, and behavioral effects of social exclusion. *Soc. Influ.* 8 217–236. 10.1080/15534510.2012.706233 27330184

[B42] Zadro L. Williams K. D. Richardson R. (2004). How low can you go? Ostracism by a computer is sufficient to lower self-reported levels of belonging, control, self-esteem, and meaningful existence. *J. Exp. Soc. Psychol.* 40 560–567. 10.1016/j.jesp.2003.11.006

