# Supplementary material for: Violated Expectations in the Cyberball Paradigm: Testing the Expectancy Account of Social Participation With ERP
Source: Front Psychol. 2018 Sep 25;9:1762. doi: 10.3389/fpsyg.2018.01762 (PMC6167485; doi:10.3389/fpsyg.2018.01762)
Supplement: Supplementary file 1 [file Data_Sheet_1.pdf]

Data Sheet 1
ERP data analysis for the event
"ball reception of the co-player (other)"

Supporting information (1)

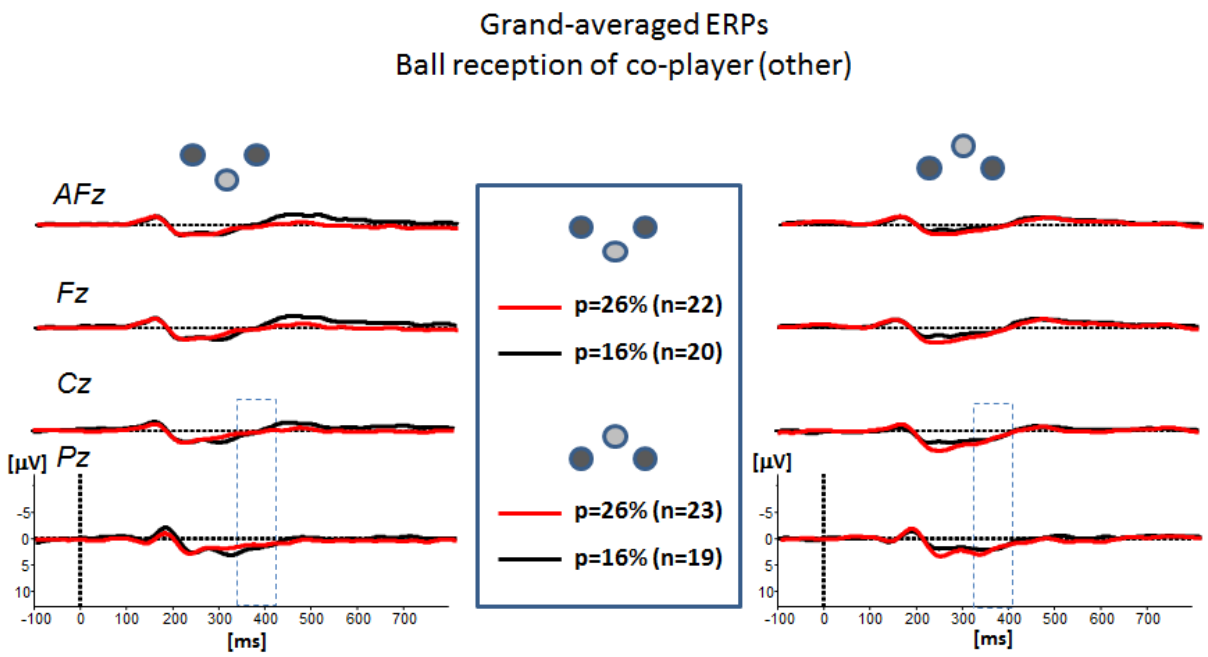

Table: ERP analysis (P3 amplitude, 340-420 ms) for the event „ball reception of others" (co-players)

Position	Probability	Mean Amplitude (uV)	SD	95% CI Lower bound	95% CI Upper bound
Superior	16%	1.144	.36	0.43	1.86
Superior	26%	1.104	.33	0.45	1.76
Inferior	16%	0.51	.35	-.19	1.21
Inferior	26%	0.87	.36	0.21	1.53

Results of the ANOVA

Factor "Position": $F(1,80) = 1.600$, $p = .210$, $\eta_p^2 = 0.020$

Factor "Probability": $F(1,80) = 0.211$, $p = .647$, $\eta_p^2 = 0.003$

Interaction of the factors: $F(1,80) = 0.333$, $p = .566$, $\eta_p^2 = 0.004$
