# Supplementary material for: Violated Expectations in the Cyberball Paradigm: Testing the Expectancy Account of Social Participation With ERP
Source: Front Psychol. 2018 Sep 25;9:1762. doi: 10.3389/fpsyg.2018.01762 (PMC6167485; doi:10.3389/fpsyg.2018.01762)
Supplement: Supplementary file 2 [file Data_Sheet_2.pdf]

Data Sheet 2:
ERP data (mean amplitude 340-420 ms) for each participant and for each electrode

Coding for the electrodes
First : Electrode position (Afz, Fz, Cz, Pz)
Second: Ball reception (s= self vs o = other player)
Third: Block position (h1=first half vs h2=second half)

File	Position	Percent	AFz_s_h1	AFz_s_h2	Fz_s_h1	Fz_s_h2	Cz_s_h1	Cz_s_h2	Pz_s_h1	Pz_s_h2
44101a	i	16	-1,6675	-1,3186	-2,5789	-1,2291	-1,0576	2,1084	0,5499	1,9019
44107a	i	16	2,3462	-1,5357	3,1318	-0,3449	5,0357	1,4697	7,3759	3,7293
44113a	i	16	9,2148	5,6781	12,5834	9,9898	12,3115	11,6543	10,4835	9,9682
44125a	i	16	0,9951	0,4787	2,8254	0,6369	4,5968	2,4179	6,6308	5,2588
44131a	i	16	-1,8888	-1,5689	-3,1718	-1,5946	0,2893	1,5504	6,0881	4,3241
44137a	i	16	4,6335	2,5503	6,3693	4,4959	9,7083	7,0089	11,8969	10,3722
44143a	i	16	0,4675	-1,9729	3,0751	-2,4378	6,8253	1,8961	11,7357	6,7907
44149a	i	16	-0,1745	2,5762	1,5886	4,1288	6,1163	6,5928	9,1367	7,9384
44150a	i	16	12,1754	10,4332	14,5365	12,7295	15,5621	15,6666	14,4730	16,0980
44171a	i	16	4,5447	2,8389	4,6740	4,9946	5,0026	9,2212	6,9028	11,9708
44202a	i	16	2,2080	-0,6923	2,5986	1,2783	6,2580	5,1779	6,9833	4,3861
44208a	i	16	0,8718	0,4484	1,9541	1,3493	4,1632	3,4608	9,3040	7,3314
44214a	i	16	3,4106	1,4316	5,2604	3,5378	5,9106	4,0431	8,5237	4,3293
44220a	i	16	5,5690	2,3108	7,5586	2,9812	8,5118	4,0106	8,7831	5,8985
44226a	i	16	4,0742	4,9438	6,3854	8,0349	8,0991	9,9822	10,6872	11,5358
44232a	i	16	-0,7851	-2,1033	-0,3317	-2,3178	2,4734	0,2516	5,2054	2,3730
44238a	i	16	4,3054	4,3262	5,3692	5,4905	10,8922	8,9188	17,7712	9,9409
44244a	i	16	-2,5674	-0,8984	-3,0438	0,1679	2,9376	5,1697	6,8354	11,9069
44265a	i	16	4,8256	9,3101	6,6456	9,8958	10,3229	9,2757	12,0449	8,1250
44266a	i	16	6,9247	8,3635	5,4171	7,1257	2,8489	2,4901	3,7407	2,7972
44101	i	26	-1,0083	-4,0482	0,3407	-3,4830	1,8643	1,2909	6,2074	3,5922
44102	i	26	0,7421	-1,7433	2,5700	-0,6863	4,3764	1,9749	5,6529	5,8075
44103	i	26	5,0027	5,1070	7,1698	5,7396	9,1650	5,2312	8,1014	6,6581
44104	i	26	1,4585	0,4735	5,0046	1,6124	10,2565	5,0216	13,1838	6,3255
44107	i	26	-3,9326	2,0432	0,7263	6,1153	9,8543	9,0310	11,3031	8,3954
44108	i	26	2,9733	6,5944	3,8853	8,9911	1,4658	10,5245	2,3295	10,3677

44110	i	26	1,6614	1,8477	4,3163	2,9808	6,7241	6,7217	6,3903	5,8918
44111	i	26	2,6171	4,7151	3,5794	7,9501	5,3416	8,0138	6,4809	6,0599
44112	i	26	2,6147	-0,9347	2,9696	-2,3220	4,9919	1,3043	7,7224	4,4370
44113	i	26	1,1206	-1,5167	2,5838	-0,7562	3,6854	0,8225	8,2513	6,2268
44114	i	26	2,8581	0,6853	4,5824	1,3924	6,8034	1,6651	7,4010	3,9032
44115	i	26	4,8784	7,9353	4,7364	7,8445	3,9067	6,7845	3,3568	4,8175
44116	i	26	0,2202	0,7214	-0,1038	-0,8716	0,9472	0,6182	4,6640	5,0126
44117	i	26	2,1536	-1,3009	2,1481	-1,4333	5,4233	3,8812	5,7092	5,5443
44119	i	26	1,1170	0,2571	0,6877	-0,3331	3,3742	1,4511	7,5389	8,3746
44122	i	26	1,8440	-1,6506	3,2766	-1,0772	3,5004	-0,1023	3,1420	-0,4109
44123	i	26	-0,7866	-2,9903	-0,3383	-2,2561	0,4244	1,0336	8,4613	4,3807
44124	i	26	0,1773	-0,7655	0,7495	-0,5067	4,6778	7,8302	14,9170	14,1618
44127	i	26	6,7815	5,4640	10,5495	7,9076	12,2170	9,8230	12,8450	10,3183
44129	i	26	0,1930	1,1407	0,1766	1,4690	3,3883	4,4200	6,5501	7,3608
44131	i	26	-1,2140	-0,3483	-0,6471	0,2304	-1,8704	0,8529	2,3136	2,5997
44133	i	26	5,2324	-1,3387	7,0555	0,0256	10,1255	3,6046	13,7322	5,8755
44309a	s	16	2,7031	3,5216	4,2350	4,5646	6,1549	7,3002	5,9755	6,6959
44315a	s	16	14,6822	10,9241	16,9719	12,1187	16,6462	13,3013	18,2687	14,4450
44321a	s	16	0,8234	2,3797	1,8938	5,8845	2,8245	11,3355	6,3070	9,6490
44327a	s	16	8,6115	11,5211	12,2990	15,5099	18,1156	21,9690	18,1058	21,6553
44333a	s	16	4,7222	3,7001	6,0966	3,8726	6,8753	6,1737	6,3437	7,1241
44339a	s	16	-1,0348	3,7005	2,4033	5,1253	6,0354	3,1562	13,3041	3,7187
44345a	s	16	8,7985	8,9170	10,1948	10,2574	13,1700	13,3499	14,3372	14,8420
44351a	s	16	10,7701	11,6219	14,1660	15,3044	16,6059	14,5995	17,9795	15,9794
44367a	s	16	0,7697	4,0791	-2,7164	4,3175	0,3061	8,7578	9,5306	11,7548
44404a	s	16	16,4478	14,8347	20,9986	18,5762	28,3444	24,4986	30,4896	25,5911
44410a	s	16	6,1572	7,3892	6,5167	8,1613	6,7119	11,3865	7,8017	10,8886
44416a	s	16	0,2936	3,5207	2,7638	8,6552	8,7738	16,1345	13,7000	23,3554
44422a	s	16	4,1112	5,2286	7,7398	10,7470	9,9853	14,6365	9,7364	15,9562
44428a	s	16	7,9211	13,0802	11,3325	15,1867	13,0072	16,1869	10,4000	11,6850
44434a	s	16	6,3164	5,3735	6,0311	4,4237	4,5312	7,9235	5,9160	11,0450
44440a	s	16	10,3729	8,2792	11,0907	7,9003	11,0883	8,1177	10,9998	8,7380
44446a	s	16	12,2318	11,2399	13,9281	12,0798	15,2801	13,1805	14,7680	13,6855

44452a	s	16	5,1693	2,1852	8,2915	3,2774	12,1440	7,0170	16,6776	14,0073
44202	s	26	2,3477	5,6891	1,7590	6,4460	4,2053	9,4927	8,6523	12,6433
44203	s	26	6,0448	3,9661	6,0747	5,0752	5,8948	4,3681	6,5189	4,2321
44205	s	26	0,4365	0,1865	-0,1328	-0,3200	-0,5451	-1,5305	4,0548	2,9703
44206	s	26	3,0849	2,3223	3,7910	2,2475	4,0834	-0,5911	7,1129	1,9271
44207	s	26	4,5917	4,0431	6,1842	4,8853	5,9007	4,5205	4,9088	4,2552
44208	s	26	1,3021	2,7444	2,2171	3,9595	3,7768	4,9756	3,4064	5,1308
44209	s	26	0,3762	0,9345	1,0585	1,4855	2,0861	3,6988	5,3419	5,8112
44211	s	26	5,7404	6,0404	7,2944	7,1059	10,9208	10,7516	10,1536	9,1043
44212	s	26	2,9519	1,9437	3,7444	3,2529	6,2653	6,7929	6,7241	9,0310
44213	s	26	-1,5422	-1,1867	0,4707	0,1445	5,5558	3,9872	8,3646	5,6204
44214	s	26	3,9272	5,2410	5,0033	5,6093	3,1242	4,4706	1,7442	5,0624
44216	s	26	9,2364	10,9706	10,8042	11,7541	9,2608	11,0771	5,9626	8,4016
44217	s	26	3,9255	-0,6250	5,0428	0,5606	7,9403	4,7696	7,1999	3,3358
44218	s	26	4,2928	2,8968	5,5990	3,1516	8,3867	4,7733	9,0775	5,4862
44219	s	26	3,4383	2,4857	2,5502	1,8684	1,2684	3,2178	1,8351	5,1396
44221	s	26	10,9093	11,8594	13,9440	14,9532	15,3918	14,5262	21,8906	18,4935
44222	s	26	4,9861	3,8359	4,0281	4,3844	1,9943	3,5379	2,2500	3,9445
44223	s	26	-0,7685	-0,9104	-1,0565	-0,6955	0,3250	2,0732	3,2869	4,8251
44224	s	26	2,0234	0,1709	1,7959	0,2660	3,1333	3,1928	5,0667	6,5067
44225	s	26	1,2595	3,7637	2,2469	4,5806	4,3602	4,5167	5,6070	4,9768
44227	s	26	-2,1990	2,6613	-1,1885	3,0219	1,0732	5,5742	4,9331	10,1455
44229	s	26	3,3539	4,9400	3,4350	5,1047	6,8921	8,5137	7,5353	8,8221
44231	s	26	2,8642	2,9932	5,1883	4,5254	7,9155	5,9017	11,6823	5,4379
44233	s	26	-2,1893	-1,1848	-3,3952	-1,5564	-1,8056	0,2307	0,1779	1,0321

Coding for the electrodes
First : Electrode position (Afz, Fz, Cz, Pz)
Second: Ball reception (s= self vs o = other player)
Third: Block position (h1=first half vs h2=second half)

File	Position	Percent	AFz_o_h1	AFz_o_h2	Fz_o_h1	Fz_o_h2	Cz_o_h1	Cz_o_h2	Pz_o_h1	Pz_o_h2
44101a	i	16	0,8891	-1,3799	0,0764	-1,8811	-0,1200	-0,1500	0,4924	-0,9545
44107a	i	16	4,0790	0,7065	3,3266	-0,5365	1,1272	-1,1598	-0,2604	-0,0164
44113a	i	16	1,7086	1,0457	1,8538	1,3394	1,9900	1,3262	5,6317	3,3116
44125a	i	16	-1,3795	0,5935	-2,3489	0,4826	-2,0993	0,9230	0,1215	2,5184
44131a	i	16	2,6942	-1,4688	3,6784	-0,8796	5,9166	2,7716	6,9300	4,4830
44137a	i	16	0,6072	1,3101	0,6613	1,3423	2,2723	0,7539	3,6121	1,4316
44143a	i	16	1,2017	1,4388	1,5877	1,9067	1,6804	1,5651	1,6733	1,9595
44149a	i	16	-0,9512	-0,2226	-0,7449	0,0430	0,8409	1,0682	1,8577	2,4760
44150a	i	16	2,0279	0,4121	1,9218	0,0494	1,2928	-0,4573	1,3225	0,2015
44171a	i	16	2,2624	1,2160	1,5383	1,1036	-1,3587	-0,0855	-1,3084	0,1937
44202a	i	16	1,3790	-1,5209	1,7805	-2,7362	2,8321	-1,6757	2,9982	0,0093
44208a	i	16	0,2569	-0,8181	0,3170	-0,7918	1,5058	-0,4564	3,6647	0,1564
44214a	i	16	0,3813	-1,7429	0,6995	-2,0172	0,6840	-2,0304	1,4746	-1,7626
44220a	i	16	-0,2652	1,3159	0,4142	2,3564	1,3778	2,6962	0,7760	1,4606
44226a	i	16	0,5036	-2,1652	0,6822	-2,5045	0,8691	-2,5054	1,6077	-1,2034
44232a	i	16	-2,1323	-1,8996	-2,6565	-2,4850	-3,5073	-3,8054	-2,2000	-3,2009
44238a	i	16	1,2846	-0,9597	1,7864	-1,3505	2,1414	-1,9477	1,6537	-3,1674
44244a	i	16	-1,2879	0,3221	-1,3530	0,6724	0,4090	2,7706	1,3627	4,1946
44265a	i	16	0,7425	1,3520	0,2417	1,2831	-2,6712	-0,9376	-3,2803	-1,9613
44266a	i	16	0,1339	-0,6019	0,6780	-1,2134	1,0871	-1,4936	2,2443	-0,1749
44101	i	26	1,9790	-1,2153	2,8566	-1,7968	5,1991	-1,1033	5,5307	0,0980
44102	i	26	-2,2432	1,5011	-1,9709	0,9932	-1,5049	0,5511	-0,8738	2,1914
44103	i	26	-1,2301	-1,3647	-0,5652	-0,5692	1,0065	1,7240	3,0444	3,0141
44104	i	26	-0,3454	-3,6001	0,0078	-3,5927	1,8526	-2,9326	2,9639	-2,9821
44107	i	26	-0,5756	-3,9552	-0,3333	-4,7237	0,4442	-2,0283	4,5118	2,7010
44108	i	26	-0,0537	-1,7847	0,1106	-2,1951	0,0145	-3,2097	0,7734	-2,6485

44110	i	26	-0,5200	-0,6428	-0,0567	0,0076	0,3240	1,8905	0,6799	2,1074
44111	i	26	0,0941	-0,9953	1,1006	-0,7687	0,6737	-0,3579	1,2645	1,2536
44112	i	26	1,2854	-0,5366	1,6407	-0,7274	1,7921	-0,3437	3,1510	3,0425
44113	i	26	-0,8582	-0,2684	-1,2807	-0,1361	-0,0332	0,3427	2,0342	-0,5810
44114	i	26	1,4036	1,6027	2,2054	1,8812	2,7235	2,2277	2,2362	3,0635
44115	i	26	-0,1874	-1,8569	-0,4569	-1,9440	-0,5766	-2,1098	-0,1011	-1,0969
44116	i	26	0,1446	0,6799	0,6878	0,9353	2,3174	0,5684	2,5843	-0,8016
44117	i	26	1,9825	-1,2059	1,2177	-1,8501	3,3837	-2,1111	3,3705	0,0579
44119	i	26	-4,3486	0,7122	-5,4222	0,7851	-4,8150	-1,7722	-0,6799	-0,7807
44122	i	26	1,1030	0,5101	1,2463	0,9336	1,6134	0,7539	0,7944	0,0437
44123	i	26	2,3149	-1,7270	3,2653	-1,8445	-0,0022	-2,1313	-2,2540	1,7733
44124	i	26	-0,6742	-4,0879	-0,5117	-3,3324	2,8489	-0,8361	8,5604	4,3716
44127	i	26	0,6964	2,2894	1,5662	2,9635	2,7496	1,8230	5,4912	3,5300
44129	i	26	1,2739	-0,1527	1,6829	-0,0283	0,9201	0,1052	0,6771	2,4870
44131	i	26	-0,8988	-3,0049	-1,7487	-3,6967	-1,8663	-3,6044	0,2871	-0,9905
44133	i	26	0,0468	-0,6141	-0,6439	-0,6394	-1,0554	1,1641	-0,4227	2,6734
44309a	s	16	-0,9746	1,2008	-0,1064	0,8086	1,9301	1,2078	4,4168	3,3786
44315a	s	16	-0,1299	0,6420	-0,0021	-0,1014	-0,0106	0,1564	0,1670	1,7078
44321a	s	16	-0,7701	-0,7554	-0,1259	-0,1807	-1,2195	-0,3333	-0,8182	-1,5386
44327a	s	16	-0,6848	-1,3781	-0,8628	-1,7537	-0,2835	-1,0069	1,4445	0,6067
44333a	s	16	3,2821	0,8622	4,0529	1,8448	3,8157	2,2160	1,7721	1,8223
44339a	s	16	0,0707	-2,1848	-0,1239	-2,6091	-1,4229	-3,7369	1,3370	-0,1555
44345a	s	16	0,2635	0,2091	0,3856	1,0940	1,0782	1,8607	1,8157	2,0857
44351a	s	16	3,8052	1,4400	4,4399	1,7719	4,1051	0,8632	5,6386	1,4868
44367a	s	16	-0,5019	0,2057	0,8005	0,9689	1,4649	-1,7653	3,3149	-0,9552
44404a	s	16	7,4138	4,4630	8,6158	5,5737	7,6277	6,7783	4,3048	6,0711
44410a	s	16	-0,8026	-2,7806	-0,7346	-3,1269	1,6351	-1,3531	4,0053	1,3911
44416a	s	16	0,0412	-0,4305	0,5935	-1,0262	2,9264	-1,2026	6,7512	0,5577
44422a	s	16	1,2358	0,9506	1,0334	0,4625	-0,1867	-1,2512	-1,8246	-1,3907
44428a	s	16	1,5547	1,3275	1,5032	0,5872	1,4099	-0,6879	1,9527	-2,0356
44434a	s	16	0,9826	-0,1826	1,2392	-0,2878	0,6915	0,4047	0,2575	2,3789
44440a	s	16	0,0806	0,9404	0,6895	1,3231	0,8372	1,7921	0,8159	2,1713
44446a	s	16	-0,3518	0,3311	1,2750	0,5825	1,7453	0,2148	2,8331	0,5442

44452a	s	16	0,4990	2,0434	0,7143	2,4894	-0,0040	0,6283	-0,6985	1,2376
44202	s	26	1,0245	-1,1939	1,3009	-0,8562	2,4531	-0,0075	3,6730	2,3692
44203	s	26	1,1754	-0,7357	1,5944	-0,4471	1,9927	-0,5527	3,9024	0,5686
44205	s	26	3,3915	1,5885	3,5654	1,1741	3,3376	-0,6308	2,6183	0,3373
44206	s	26	0,2777	-0,6515	0,1380	-0,3922	-1,0876	-0,1769	0,1766	0,4283
44207	s	26	-2,4240	-1,2187	-3,1835	-2,3458	-2,5508	-2,5739	-2,0636	-0,7537
44208	s	26	1,1176	0,0506	1,3189	-0,0034	2,0389	-0,7165	2,1372	-1,1030
44209	s	26	-1,7862	-0,9199	-1,3287	-0,5895	-1,7633	0,1735	-2,5042	1,0672
44211	s	26	0,1407	-0,9026	0,4166	-0,9055	1,6485	0,2130	1,4807	-0,5102
44212	s	26	1,0335	1,9727	1,1219	2,8185	2,1627	3,4563	2,6479	3,5774
44213	s	26	-3,7328	-1,0019	-4,1283	-0,9413	-4,7991	-0,4983	-0,6679	1,2904
44214	s	26	-0,7163	0,0034	-0,5112	-0,0019	1,0276	0,8525	-1,3234	2,5391
44216	s	26	3,6214	3,3383	5,0465	4,3076	5,4296	4,0978	5,0915	2,4770
44217	s	26	-0,1701	-0,3025	1,3019	-0,4405	2,7468	-1,0242	3,6969	0,5550
44218	s	26	1,2349	0,7348	2,3493	1,4683	2,4385	1,7034	1,5624	1,4151
44219	s	26	-0,5139	1,4665	-0,0224	2,1860	0,7329	2,8865	1,3616	3,5480
44221	s	26	-0,1413	-1,9545	-0,6358	-2,1279	-2,5009	-2,3820	-2,5637	-2,0137
44222	s	26	0,3405	-2,4478	0,5042	-2,1311	0,6730	0,8214	1,0650	2,3021
44223	s	26	-4,6154	3,0694	-4,9183	3,1529	-3,7451	2,1593	-3,2968	1,1295
44224	s	26	0,3872	1,3332	1,2649	1,1640	2,3825	1,1223	1,6450	1,6637
44225	s	26	0,9726	1,4351	3,5197	0,5310	2,4035	-1,3504	3,2006	-2,8732
44227	s	26	-4,7370	2,6728	-3,7503	3,4512	0,2846	4,4742	1,2835	4,3091
44229	s	26	2,8811	0,6302	3,0313	0,6423	4,5057	1,4111	6,5812	2,1451
44231	s	26	3,0331	-0,6324	3,8220	-0,3486	5,0172	0,1823	3,7523	-1,2446
44233	s	26	0,8784	-0,8841	-0,0996	-1,5401	-0,3289	-0,3693	0,3203	-1,3876

Coding for the electrodes
First : Electrode position (Fz, Cz, Pz), CP=mean of Cz and Pz
Second: diff=difference(self-other) , or split position (half 1 vs half2)
Third: Block position (h1=first half vs h2=second half)

File	Position	Percent	fz_diff	cz_diff	pz_diff	**cp__diff**	**cp_half1**	**cp_half2**
44101a	i	16	2,09	3,06	2,27	2,67	-0,44	2,56
44107a	i	16	0,77	4,15	6,49	5,32	5,77	3,19
44113a	i	16	10,20	10,27	5,76	8,01	7,59	8,49
44125a	i	16	1,88	1,91	1,26	1,59	6,60	2,12
44131a	i	16	-2,46	-0,99	2,67	0,84	-3,23	-0,69
44137a	i	16	4,03	6,12	7,85	6,99	7,86	7,60
44143a	i	16	2,05	4,95	6,90	5,92	7,60	2,58
44149a	i	16	3,25	5,79	7,08	6,43	6,28	5,49
44150a	i	16	13,06	15,24	14,49	14,86	13,71	16,01
44171a	i	16	3,77	7,65	10,13	8,89	7,29	10,54
44202a	i	16	1,87	4,88	4,30	4,59	3,71	5,62
44208a	i	16	3,01	4,36	8,38	6,37	4,15	5,55
44214a	i	16	5,93	6,67	6,68	6,68	6,14	6,08
44220a	i	16	3,79	4,18	6,25	5,21	7,57	2,88
44226a	i	16	8,22	10,10	11,12	10,61	8,15	12,61
44232a	i	16	3,07	7,70	8,91	8,30	6,69	4,82
44238a	i	16	4,69	9,45	14,50	11,98	12,43	11,99
44244a	i	16	0,36	3,48	6,89	5,19	4,00	5,06
44265a	i	16	8,79	12,68	13,79	13,24	14,16	10,15
44266a	i	16	6,10	2,86	2,32	2,59	1,63	3,48
44101	i	26	-1,40	0,85	3,01	1,93	-1,33	2,94
44102	i	26	1,27	3,49	5,02	4,25	6,20	2,52
44103	i	26	6,65	5,50	4,10	4,80	6,61	3,58
44104	i	26	4,58	7,77	9,29	8,53	9,31	8,63
44107	i	26	4,96	8,45	5,01	6,73	8,10	8,38
44108	i	26	7,47	7,13	6,85	6,99	1,50	13,38

44110	i	26	3,50	5,38	4,59	4,99	6,06	4,31
44111	i	26	5,78	6,25	4,73	5,49	4,94	6,59
44112	i	26	0,59	3,28	3,68	3,48	3,89	1,52
44113	i	26	2,25	2,01	6,38	4,20	4,97	3,64
44114	i	26	1,39	2,17	3,24	2,71	4,62	0,14
44115	i	26	7,81	7,13	5,06	6,09	3,97	7,40
44116	i	26	-1,41	-0,69	3,82	1,56	0,35	2,93
44117	i	26	-0,02	3,76	3,64	3,70	2,19	5,74
44119	i	26	3,39	6,63	9,98	8,31	8,20	6,19
44122	i	26	-0,13	0,48	0,98	0,73	2,12	-0,66
44123	i	26	-1,97	1,81	6,63	4,22	5,57	2,89
44124	i	26	4,18	6,72	9,56	8,14	4,09	9,23
44127	i	26	6,75	9,00	7,20	8,10	8,41	7,39
44129	i	26	-0,72	3,58	5,44	4,51	4,17	4,59
44131	i	26	0,80	0,59	1,51	1,05	1,01	4,02
44133	i	26	3,81	6,78	8,85	7,82	12,67	2,82
44309a	s	16	2,09	-0,04	6,05	3,00	2,89	4,70
44315a	s	16	3,11	3,85	1,20	2,52	17,38	12,94
44321a	s	16	11,96	12,98	13,47	13,22	5,58	11,43
44327a	s	16	3,96	7,56	8,79	8,18	17,53	22,01
44333a	s	16	14,66	19,77	18,56	19,17	3,82	4,63
44339a	s	16	1,96	3,67	4,90	4,29	9,71	5,38
44345a	s	16	4,68	7,00	7,81	7,41	12,31	12,12
44351a	s	16	10,72	13,41	14,87	14,14	12,42	14,11
44367a	s	16	11,63	14,23	15,10	14,66	2,53	11,62
44404a	s	16	-0,96	4,34	9,41	6,88	23,45	18,62
44410a	s	16	15,52	21,02	22,37	21,69	4,44	11,12
44416a	s	16	8,49	8,48	6,23	7,36	6,40	20,07
44422a	s	16	7,08	12,98	16,69	14,83	10,87	16,62
44428a	s	16	9,86	13,02	14,81	13,91	10,02	15,30
44434a	s	16	10,77	13,42	10,96	12,19	4,75	8,09
44440a	s	16	5,26	5,98	7,23	6,60	10,22	6,45
44446a	s	16	9,34	9,01	8,63	8,82	12,73	13,05

44452a	s	16	8,00	7,62	6,79	7,20	14,76	9,58
44202	s	26	3,71	8,20	13,59	10,90	3,37	9,89
44203	s	26	4,29	5,50	7,59	6,54	3,26	4,29
44205	s	26	4,60	4,27	3,26	3,76	-1,22	0,87
44206	s	26	-1,65	-1,20	2,67	0,74	6,05	0,54
44207	s	26	3,95	3,46	5,31	4,39	7,71	6,05
44208	s	26	7,51	7,27	5,50	6,39	1,50	5,96
44209	s	26	1,72	3,25	3,83	3,54	5,85	4,13
44211	s	26	2,04	3,46	5,83	4,65	8,97	10,08
44212	s	26	5,41	8,99	8,85	8,92	4,09	4,40
44213	s	26	1,93	4,07	5,29	4,68	9,69	4,41
44214	s	26	3,23	8,26	7,85	8,06	2,58	3,07
44216	s	26	6,58	3,98	3,87	3,93	2,35	6,45
44217	s	26	6,13	5,03	3,03	4,03	4,35	4,29
44218	s	26	3,25	5,89	3,46	4,67	6,73	3,57
44219	s	26	3,03	4,98	6,19	5,59	0,50	0,96
44221	s	26	1,40	0,68	1,05	0,86	21,17	18,71
44222	s	26	15,72	17,97	22,82	20,39	1,25	2,18
44223	s	26	5,33	2,38	1,75	2,06	5,33	1,80
44224	s	26	1,02	2,45	5,31	3,88	2,09	3,46
44225	s	26	0,70	2,44	4,57	3,50	2,18	6,86
44227	s	26	3,47	5,47	5,03	5,25	2,22	3,47
44229	s	26	0,02	2,03	5,43	3,73	1,67	6,89
44231	s	26	3,06	5,52	4,00	4,76	5,41	6,20
44233	s	26	3,46	4,38	6,42	5,40	-0,81	1,51
