# Supplementary material for: Violated Expectations in the Cyberball Paradigm: Testing the Expectancy Account of Social Participation With ERP
Source: Front Psychol. 2018 Sep 25;9:1762. doi: 10.3389/fpsyg.2018.01762 (PMC6167485; doi:10.3389/fpsyg.2018.01762)
Supplement: Supplementary file 3 [file Data_Sheet_3.pdf]

Data Sheet 3: NTQ data for each participant for each NTQ scale, and for the negative mood scale

Participant	Vertical Position	Ball Reception (relative)	Estimated Ball Reception (relative)	NTQ Belonging	NTQ Self esteem	NTQ Meaningful Existence	NTQ Control	NTQ MEAN	Negative Mood Scale
44303	superior	0,16	0,15	1,6670	2,0000	4,0000	2,0000	2,4168	12,0000
44309	superior	0,16	0,17	3,6670	2,3330	3,0000	1,3330	2,5833	11,0000
44315	superior	0,16	0,06	2,6670	3,0000	4,6670	2,0000	3,0835	11,5000
44321	superior	0,16	0,15	3,6670	3,3330	3,0000	1,6670	2,9168	9,5000
44327	superior	0,16	0,05	2,0000	3,0000	1,6670	2,3330	2,2500	12,0000
44333	superior	0,16	0,20	4,3330	4,3330	3,3330	3,3330	3,8330	8,0000
44339	superior	0,16	0,07	1,0000	1,0000	1,0000	1,0000	1,0000	17,5000
44345	superior	0,16	0,10	2,3330	2,0000	3,6670	1,0000	2,2500	11,5000
44351	superior	0,16	0,10	2,3330	2,6670	3,3330	1,6670	2,5000	16,0000
44410	superior	0,16	0,13	1,6670	2,0000	2,3330	1,6670	1,9168	14,5000
44416	superior	0,16	0,25	2,3330	2,6670	3,6670	1,0000	2,4168	12,5000
44422	superior	0,16	0,15	2,0000	2,6670	3,6670	1,0000	2,3335	10,0000
44428	superior	0,16	0,05	1,3330	1,3330	1,3330	2,3330	1,5830	11,5000
44434	superior	0,16	0,15	4,0000	4,0000	1,6670	2,6670	3,0835	5,0000
44440	superior	0,16	0,22	2,3330	2,0000	5,0000	1,3330	2,6665	11,5000
44446	superior	0,16	0,21	3,0000	2,6670	2,3330	2,6670	2,6668	9,5000
44452	superior	0,16	0,15	3,6670	2,3330	3,6670	1,3330	2,7500	8,0000
44367	superior	0,16	0,25	3,3000	2,7000	5,0000	1,0000	3,0000	10,5000
44404	superior	0,16	0,10	3,3300	1,3300	2,7000	1,0000	2,0900	13,5000
44202	superior	0,27	0,20	3,3333	2,6667	5,0000	2,3333	3,3333	7,5000
44203	superior	0,27	0,15	2,3333	2,3333	3,3333	1,0000	2,2500	15,5000
44205	superior	0,27	0,25	4,3333	3,3333	5,0000	1,3333	3,5000	5,5000
44206	superior	0,27	0,25	4,3333	4,0000	4,6667	2,3333	3,8333	7,0000
44207	superior	0,27	0,29	4,6667	3,0000	4,6667	1,3333	3,4167	8,5000
44208	superior	0,27	0,25	2,0000	2,3333	1,6667	3,0000	2,2500	13,5000
44209	superior	0,27	0,20	4,6667	4,0000	5,0000	2,0000	3,9167	4,5000
44211	superior	0,27	0,10	3,6667	3,6667	3,3333	1,3333	3,0000	10,0000
44212	superior	0,27	0,25	3,6667	3,3333	5,0000	2,6667	3,6667	8,0000

44213	superior	0,27	0,12	4,0000	3,3333	3,3333	4,0000	3,6667	8,5000
44214	superior	0,27	0,20	2,3333	2,3333	4,0000	1,6667	2,5833	12,5000
44216	superior	0,27	0,23	2,6667	2,6667	2,3333	1,6667	2,3333	15,5000
44217	superior	0,27	0,25	2,6667	3,3333	4,3333	1,3333	2,9167	8,0000
44218	superior	0,27	0,30	4,6667	5,0000	5,0000	1,6667	4,0833	4,5000
44219	superior	0,27	0,60	2,6667	1,6667	5,0000	1,3333	2,6667	14,5000
44221	superior	0,27	0,25	4,0000	2,0000	4,6667	1,6667	3,0833	10,5000
44222	superior	0,27	0,25	3,3333	4,3333	5,0000	3,6667	4,0833	6,0000
44223	superior	0,27	0,35	3,6667	3,0000	3,0000	1,3333	2,7500	9,0000
44224	superior	0,27	0,33	3,3333	4,6667	2,6667	8,5000	4,7917	8,5000
44225	superior	0,27	0,30	4,0000	4,0000	5,0000	1,6667	3,6667	6,0000
44227	superior	0,27	0,30	3,6667	3,6667	4,0000	2,3333	3,4167	10,0000
44229	superior	0,27	0,20	3,6667	2,6667	4,6667	2,0000	3,2500	11,0000
44231	superior	0,27	0,18	2,3333	2,6667	2,6667	1,3333	2,2500	13,5000
44101	inferior	0,16	0,10	3,6670	2,3330	5,0000	1,3330	3,0833	8,0000
44107	inferior	0,16	0,10	2,3330	2,6670	3,0000	2,3330	2,5833	9,5000
44113	inferior	0,16	0,10	2,0000	3,0000	2,6670	1,6670	2,3335	11,5000
44125	inferior	0,16	0,15	3,0000	3,3330	5,0000	2,3330	3,4165	7,0000
44131	inferior	0,16	0,15	3,0000	2,0000	2,6670	1,3330	2,2500	13,5000
44137	inferior	0,16	0,10	4,0000	3,6670	4,0000	2,3330	3,5000	7,0000
44143	inferior	0,16	0,20	1,3330	2,0000	3,3330	1,6670	2,0833	11,0000
44149	inferior	0,16	0,08	2,3330	2,6670	4,3330	1,6670	2,7500	9,0000
44150	inferior	0,16	0,05	2,0000	3,0000	2,0000	3,0000	2,5000	7,5000
44202	inferior	0,16	0,30	2,0000	4,3000	2,0000	3,0000	2,8250	9,0000
44171	inferior	0,16	0,10	5,0000	3,3300	3,7000	2,7000	3,6825	6,5000
44208	inferior	0,16	0,20	1,6670	3,0000	2,0000	2,0000	2,1668	11,5000
44214	inferior	0,16	0,15	2,6670	3,3330	3,6670	2,6670	3,0835	6,5000
44220	inferior	0,16	0,15	3,6670	2,6670	2,6670	3,6670	3,1670	12,0000
44226	inferior	0,16	0,09	2,3330	2,3330	2,3330	2,0000	2,2498	9,5000
44232	inferior	0,16	0,33	5,0000	5,0000	5,0000	2,6670	4,4168	4,0000
44238	inferior	0,16	0,15	4,0000	3,6670	3,0000	1,6670	3,0835	10,0000
44244	inferior	0,16	0,10	3,0000	2,3330	2,6670	1,3330	2,3333	13,0000
44265	inferior	0,16	0,05	4,0000	5,0000	5,0000	2,6670	4,1668	4,5000

44266	inferior	0,16	0,02	3,6670	3,0000	5,0000	1,0000	3,1668	7,5000
44101	inferior	0,27	0,20	4,0000	3,6667	4,0000	2,3333	3,5000	8,0000
44102	inferior	0,27	0,33	3,6667	4,3333	4,6667	2,0000	3,6667	8,0000
44103	inferior	0,27	0,30	3,6667	3,0000	5,0000	2,6667	3,5833	10,0000
44104	inferior	0,27	0,40	4,3333	4,6667	5,0000	3,0000	4,2500	5,0000
44107	inferior	0,27	0,11	2,3333	1,6667	3,0000	1,6667	2,1667	13,0000
44108	inferior	0,27	0,33	3,6667	3,6667	4,6667	3,6667	3,9167	8,0000
44110	inferior	0,27	0,25	3,3333	4,3333	4,6667	3,3333	3,9167	6,5000
44111	inferior	0,27	0,55	4,0000	4,6667	5,0000	2,3333	4,0000	8,0000
44112	inferior	0,27	0,25	3,6667	3,6667	4,6667	1,6667	3,4167	12,0000
44113	inferior	0,27	0,30	4,6667	3,6667	4,6667	3,3333	4,0833	9,5000
44114	inferior	0,27	0,15	2,6667	3,3333	4,3333	2,6667	3,2500	10,5000
44115	inferior	0,27	0,30	3,6667	3,0000	4,6667	2,3333	3,4167	7,0000
44116	inferior	0,27	0,30	4,3333	3,6667	5,0000	1,3333	3,5833	6,0000
44117	inferior	0,27	0,15	2,3333	3,3333	4,6667	1,6667	3,0000	12,5000
44119	inferior	0,27	0,19	3,6667	4,0000	5,0000	3,0000	3,9167	4,5000
44122	inferior	0,27	0,28	3,0000	3,0000	5,0000	1,0000	3,0000	6,0000
44123	inferior	0,27	0,18	3,3333	3,6667	4,6667	1,6667	3,3333	5,0000
44124	inferior	0,27	0,25	3,3333	3,0000	4,6667	1,6667	3,1667	8,5000
44127	inferior	0,27	0,28	2,3333	3,0000	5,0000	2,0000	3,0833	10,0000
44129	inferior	0,27	0,10	3,0000	2,6667	3,6667	1,3333	2,6667	11,0000
44131	inferior	0,27	0,25	4,3333	3,3333	4,6667	2,6667	3,7500	8,5000
44133	inferior	0,27	0,27	5,0000	3,6667	4,3333	3,0000	4,0000	6,0000
