# Supplementary material for: Violated Expectations in the Cyberball Paradigm: Testing the Expectancy Account of Social Participation With ERP
Source: Front Psychol. 2018 Sep 25;9:1762. doi: 10.3389/fpsyg.2018.01762 (PMC6167485; doi:10.3389/fpsyg.2018.01762)
Supplement: Supplementary file 4 [file Data_Sheet_4.pdf]

Data Sheet 4:
- Mean and median response times
- Mean ocular response (vEOG and hEOG) in the time range 340-420 ms

Subject = Subject-ID
prob = Probability (16% and 26% ball reception)
posit = Position (u=inferior vs o=superior)
heog= mean amplitude of hEOG in the time range 340-420
veog = mean amplitude of vEOG in the time range 340-420
heogs_h1 and hEOGs_h2 = mean amplitudes in the first and second half
veogs_h1 and vEOGs_h2 = mean amplitudes in the first and second half
RT_mean = Participant's mean decision time (ball throw)
RT_median = Median of the participant's decision time (ball throw)
RT_SD = Standard deviation of the participant's decision time (ball throw)

Subject	prob	posit	heog	veog	hEOGs_h1	hEOGs_h2	vEOGs_h1	vEOGs_h2	RT_mean	RT_median	RT_SD
44101a	16	u	05	-10	0.003	0.007	-0.009	-0.010	927	701	459
44107a	16	u	-02	-03	0.001	-0.004	0.000	-0.006	987	924	269
44113a	16	u	-02	04	-0.001	-0.003	0.005	0.002	696	621	435
44125a	16	u	-01	-03	0.001	-0.004	-0.005	-0.001	1.605	1.112	1.329
44131a	16	u	-15	-05	-0.005	-0.025	-0.003	-0.006	805	725	411
44137a	16	u	-01	05	0.000	-0.002	0.006	0.004	1.556	1.284	1.103
44143a	16	u	00	-08	-0.002	0.003	-0.010	-0.006	630	511	472
44149a	16	u	03	00	0.003	0.003	0.000	0.000	1.112	1.039	306
44150a	16	u	-01	04	-0.005	0.002	0.004	0.005	795	691	335
44171a	16	u	04	04	0.002	0.006	0.005	0.002	831	778	455
44202a	16	u	-03	-01	-0.003	-0.003	0.001	-0.003	2.272	1.613	1.880
44208a	16	u	-01	-09	-0.004	0.001	-0.006	-0.012	625	567	289
44214a	16	u	01	-07	0.002	-0.001	-0.005	-0.009	749	667	375
44220a	16	u	00	03	-0.001	0.000	0.003	0.002	496	458	226
44226a	16	u	02	-01	0.001	0.003	0.000	-0.002	1.026	918	405
44232a	16	u	-04	-04	-0.008	-0.001	-0.003	-0.004	1.895	1.341	1.258
44238a	16	u	00	-01	-0.001	0.001	-0.001	-0.001	867	755	469
44244a	16	u	-02	-07	0.005	-0.008	-0.004	-0.010	907	867	236
44265a	16	u	09	06	0.011	0.007	0.003	0.009	789	713	369
44266a	16	u	01	04	-0.001	0.002	0.003	0.005	976	807	793
44101.	26	u	01	-06	0.001	0.000	-0.002	-0.010	722	627	400
44102.	26	u	00	-08	0.001	-0.001	-0.004	-0.013	741	690	215
44103.	26	u	02	06	-0.001	0.004	0.006	0.005	680	512	529

44104.	26 u	00	-05	0.000	0.000	-0.009	0.000	516	487	169
44107.	26 u	-02	-12	0.004	-0.008	-0.014	-0.009	808	743	305
44108.	26 u	-01	01	-0.001	0.000	0.000	0.002	902	760	469
44110.	26 u	-01	-04	-0.001	0.000	-0.006	-0.001	558	532	162
44111.	26 u	01	03	0.000	0.001	0.003	0.004	1.128	819	775
44112.	26 u	04	02	0.007	0.001	0.003	0.001	1.010	867	404
44113.	26 u	01	-01	0.000	0.003	0.000	-0.001	634	497	369
44114.	26 u	01	02	0.001	0.000	0.003	0.002	549	491	268
44115.	26 u	02	05	0.003	0.001	0.004	0.006	1.162	915	723
44116.	26 u	-02	-01	-0.002	-0.002	-0.001	0.000	677	646	158
44117.	26 u	-03	-03	-0.004	-0.003	0.003	-0.009	716	605	565
44119.	26 u	00	01	0.000	0.000	0.002	0.000	867	867	369
44122.	26 u	03	-05	0.006	-0.001	-0.003	-0.007	984	878	417
44123.	26 u	00	-01	-0.002	0.001	0.000	-0.003	975	931	309
44124.	26 u	-01	-13	-0.002	0.000	-0.013	-0.013	842	725	492
44127.	26 u	13	-05	0.018	0.008	-0.004	-0.005	694	605	247
44129.	26 u	-03	02	-0.001	-0.006	0.003	0.002	950	713	861
44131.	26 u	00	00	0.002	-0.002	-0.002	0.002	936	873	303
44133.	26 u	01	03	0.000	0.002	0.003	0.003	546	523	170
44309a	16 o	-06	05	-0.001	-0.010	0.004	0.006	1.109	756	2.232
44315a	16 o	-13	15	-0.012	-0.014	0.019	0.011	1.761	1.612	694
44321a	16 o	01	02	-0.001	0.003	0.002	0.002	1.151	1.077	351
44327a	16 o	-01	05	0.000	-0.001	0.003	0.007	3.527	3.080	2.293
44333a	16 o	-01	02	0.001	-0.002	0.002	0.002	409	368	133
44339a	16 o	01	02	0.001	0.000	0.000	0.004	543	517	138
44345a	16 o	-10	13	-0.006	-0.014	0.012	0.013	1.123	1.018	561
44351a	16 o	03	09	0.004	0.002	0.010	0.008	1.123	1.018	561
44367a	16 o	-02	07	0.000	-0.004	0.008	0.006	759	713	213
44404a	16 o	-01	21	0.001	-0.003	0.023	0.019	623	593	247
44410a	16 o	-07	09	-0.006	-0.008	0.008	0.009	1.165	989	555
44416a	16 o	-03	08	-0.003	-0.003	0.009	0.007	1.003	936	311
44422a	16 o	-01	08	-0.002	-0.001	0.006	0.009	880	684	607
44428a	16 o	-01	10	0.001	-0.002	0.008	0.013	917	851	328
44434a	16 o	01	04	0.001	0.001	0.004	0.005	680	595	340
44440a	16 o	01	18	0.007	-0.004	0.018	0.018	1.060	876	594

44446a	16	o	07	19	0.005	0.009	0.018	0.019	1.078	965	435
44452a	16	o	02	09	0.004	0.001	0.007	0.010	694	676	203
44202.	26	o	01	08	0.000	0.001	0.004	0.011	1.334	869	1.016
44203.	26	o	01	06	-0.001	0.003	0.007	0.004	771	538	597
44205.	26	o	01	05	0.003	-0.002	0.005	0.006	560	508	206
44206.	26	o	-02	09	-0.004	0.000	0.009	0.009	1.398	860	1.550
44207.	26	o	03	08	0.002	0.004	0.006	0.009	920	755	566
44208.	26	o	-01	06	-0.002	-0.001	0.003	0.009	870	714	457
44209.	26	o	00	01	0.000	0.000	0.002	0.001	852	801	274
44211.	26	o	00	11	0.003	-0.003	0.011	0.010	652	634	128
44212.	26	o	01	03	0.000	0.001	0.004	0.002	427	379	251
44213.	26	o	00	02	0.005	-0.005	0.004	0.001	810	652	848
44214.	26	o	-01	04	0.000	-0.001	0.004	0.004	1.315	895	832
44216.	26	o	01	02	-0.003	0.004	0.002	0.002	1.671	1.284	1.014
44217.	26	o	00	06	0.000	0.000	0.007	0.005	644	657	155
44218.	26	o	01	02	0.000	0.001	0.004	0.001	593	508	320
44219.	26	o	-02	06	-0.001	-0.003	0.006	0.005	877	708	371
44221.	26	o	01	15	-0.001	0.003	0.015	0.015	1.296	1.112	564
44222.	26	o	04	04	0.000	0.007	0.004	0.005	843	754	616
44223.	26	o	02	00	-0.002	0.006	0.000	0.000	1.432	1.241	708
44224.	26	o	02	04	0.004	0.000	0.006	0.002	2.365	1.622	2.239
44225.	26	o	03	08	0.002	0.004	0.011	0.006	920	714	251
44227.	26	o	-01	08	0.002	-0.005	0.003	0.013	1.002	942	365
44229.	26	o	00	05	-0.001	0.001	0.005	0.006	1.180	907	1.102
44231.	26	o	02	10	0.000	0.005	0.011	0.008	1.004	948	436
