# Supplementary material for: Violated Expectations in the Cyberball Paradigm: Testing the Expectancy Account of Social Participation With ERP
Source: Front Psychol. 2018 Sep 25;9:1762. doi: 10.3389/fpsyg.2018.01762 (PMC6167485; doi:10.3389/fpsyg.2018.01762)
Supplement: Supplementary file 5 [file Data_Sheet_5.pdf]

Data Sheet 5:
- Analysis of the effect of experimental manipulation on the vEOG level
- Analysis of the vEOG and ERP effects in the control time range (240-320 ms)

vEOG Analysis (340 – 420 ms)

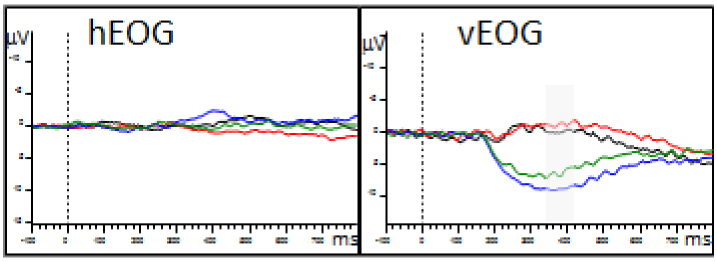

Probability	Position	Color (Figure)	Mean, SEM	Confidence Interval
16%	Superior	Blue	9.05, 1.15	6.76, 11.32
16%	Inferior	Green	-1.39, 1.09	-3.55, 0.77
26%	Superior	Red	5.82, 1.01	3.81, 7.84
26%	Inferior	Black	-1.66, 1.04	-3.81, 0.40

Main effects

Factor ‘position‘: $F(1,80) = 67.73$, $p < 0.001$, $\eta_p^2=0.458$

Factor ‘probability’: $F(1,80) = 2.18$, $p = 0.144$, $\eta_p^2=0.027$

Factor ‘half’: $F(1,80) = 0.906$, $p = 0.344$, $\eta_p^2=0.011$

Interaction with factor ‘position’

‘Probability’ x ‘Position’ $F(1,80) = 1.489$, $p = 0.226$, $\eta_p^2=0.018$

‘Half’ x ‘Position’ $F(1,80) = 1.899$, $p = 0.172$, $\eta_p^2 = 0.023$

Effect on vEOG on P3 expression

I. Analysis of the vEOG und P3 signal – separated for the first and second halves of each block

Direct comparison

Effects of verticality on EOG and P3 –separated the first and second half

	vEOG (340-420 ms)	P3 (340-420 ms)
First half	$F(1,81)=51.82$<br>$p < 0.001$<br>$\eta_p^2 = 0.387$	$F(1,81)=2.426$<br>$p = 0.123$<br>$\eta_p^2 = 0.029$
Second half	$F(1,81)=61.82$<br>$p < 0.001$<br>$\eta_p^2 = 0.428$	$F(1,81)=9.702$<br>$p = 0.003$<br>$\eta_p^2 = 0.107$

P3 effect of verticality, but not vEOG, is restricted to the second half

II. Analysis of the time range preceding the P3 effect (240 – 320 ms)

Signal	Position	Mean, SEM	Confidence Interval
vEOG	Superior	6.66, 0.68	5.31, 8.02
vEOG	Inferior	-0.92, 0.67	-2.25, 0.46
ERP (Cz, Pz)	Superior	6.55, 0.53	5.50, 7.63
ERP (Cz, Pz)	Inferior	5.96, 0.52	5.50, 7.60

Main effects of the experimental factor 'position':

vEOG $F(1,80) = 63.09$, $p < 0.001$, $\eta_p^2=0.438$

ERP $F(1,80) = 0.65$, $p = 0.422$, $\eta_p^2=0.008$

The vEOG effect of verticality is already highly expressed in the a previous time range (240-320ms), but cannot be observed in the corresponding time rage in the ERPs
