# Supplementary material for: Violated Expectations in the Cyberball Paradigm: Testing the Expectancy Account of Social Participation With ERP
Source: Front Psychol. 2018 Sep 25;9:1762. doi: 10.3389/fpsyg.2018.01762 (PMC6167485; doi:10.3389/fpsyg.2018.01762)
Supplement: Supplementary file 6 [file Data_Sheet_6.pdf]

Data Sheet 6:
Mean vEOG and ERP activity level in a control time range (240-320 ms)

Mean amplitude 240 - 320 ms
probit = Probability (16% and 26% ball reception)
posi = Position (i=inferior, s=superior)

File	probit	posi	hEOG	vEOG	Afz	Fz
44101a	16	i	4,62562	-6,013947	6,563407	6,49294
44107a	16	i	2,470085	-0,782308	4,84512	6,648384
44113a	16	i	-0,185119	0,812208	2,957344	5,162797
44125a	16	i	-0,15389	-4,896694	-0,620261	-0,560958
44131a	16	i	-18,143282	-5,936939	1,831248	2,822301
44137a	16	i	-0,671491	4,360088	2,166231	3,440538
44143a	16	i	0,739454	0,642461	7,212168	10,021331
44149a	16	i	3,647356	0,85063	1,130564	2,141335
44150a	16	i	0,06448	2,769869	6,978143	9,434182
44171a	16	i	2,709677	3,895566	2,569648	1,896065
44202a	16	i	-0,848642	-4,513351	2,442609	4,24339
44208a	16	i	-0,526944	-7,96435	0,284343	1,445243
44214a	16	i	-2,199153	-2,69421	4,589527	7,038332
44220a	16	i	0,568374	3,159481	6,04353	6,034164
44226a	16	i	4,769535	-0,075436	7,226327	9,501105
44232a	16	i	-4,919672	-4,091102	3,955774	5,977866
44238a	16	i	1,430747	-1,783039	2,261648	3,723475
44244a	16	i	0,317682	-0,573997	6,177756	8,469547
44265a	16	i	8,524367	1,525269	2,658661	3,48138
44266a	16	i	-0,154146	3,454328	5,041362	4,294948
44309a	16	s	-4,524242	6,532959	8,248599	9,182265
44315a	16	s	-9,075727	10,069673	4,459991	5,921142
44321a	16	s	2,475316	3,073305	6,858834	12,167142
44327a	16	s	0,902836	2,42347	3,148001	4,290122
44333a	16	s	-0,219272	2,94618	2,742561	1,879719
44339a	16	s	-0,005363	2,077201	1,536886	4,639957
44345a	16	s	-5,914874	9,584447	7,306672	8,100181
44351a	16	s	2,147977	7,276991	2,487633	1,635706
44367a	16	s	-0,18398	2,878143	2,678952	3,85039
44404a	16	s	2,229256	21,311134	11,727038	14,470288
44410a	16	s	-4,877044	8,807775	6,421032	8,547272
44416a	16	s	1,142503	8,113374	5,063102	7,363142
44422a	16	s	-5,878853	8,409376	2,428593	2,964137
44428a	16	s	0,680677	7,662929	7,112546	9,907628
44434a	16	s	0,153124	4,226674	3,66059	3,373087
44440a	16	s	5,466745	15,507054	9,326583	8,879231
44446a	16	s	5,452617	7,884874	3,361473	4,632149
44452a	16	s	3,557453	8,238376	2,006155	2,754379
44101	26	i	-0,176069	-1,211596	0,624564	0,704207
44102	26	i	-1,581945	6,166299	1,998189	3,040628
44103	26	i	2,899645	-4,693508	3,391752	4,847771
44104	26	i	-0,338042	2,651702	3,072793	4,391393
44107	26	i	-1,35622	7,943855	0,881685	3,276805

44108	26	i	-1,649598	-0,725952	3,48658	4,20536
44110	26	i	0,073042	3,310038	3,125812	5,510285
44111	26	i	-0,428318	-2,080285	2,627677	3,794518
44112	26	i	1,23843	-3,182875	8,722087	11,708531
44113	26	i	-0,202197	-2,612968	4,601185	5,746086
44114	26	i	0,804993	-1,915503	2,011747	3,122256
44115	26	i	0,977475	-5,919602	8,376284	10,523059
44116	26	i	-3,536509	-0,274774	3,10259	5,945383
44117	26	i	-0,108315	0,29397	3,728421	6,530481
44119	26	i	-1,825759	-2,604434	5,640962	6,924335
44122	26	i	1,721108	2,997308	3,600548	4,512596
44123	26	i	-0,65628	1,117107	-0,6069	-0,928837
44124	26	i	-1,593517	15,714917	1,103644	4,218954
44127	26	i	8,026655	7,258441	0,800717	2,159075
44129	26	i	-2,677842	-0,731669	4,265172	5,32703
44131	26	i	-0,696176	1,281373	0,414498	1,589179
44133	26	i	0,422781	-2,042945	2,638169	4,561059
44202	26	s	-0,002758	-7,317029	5,940818	8,674995
44203	26	s	1,001071	-3,91497	7,499176	8,427668
44205	26	s	-0,119619	-6,505583	2,993478	4,467964
44206	26	s	-0,828142	-8,187873	2,420877	3,382719
44207	26	s	2,614599	-6,145211	3,024318	3,584734
44208	26	s	-0,468148	-6,020706	5,14763	5,879761
44209	26	s	0,064883	-1,474148	1,278669	0,882871
44211	26	s	-2,654409	-9,576225	5,532843	5,983075
44212	26	s	1,111212	-0,804761	-0,31914	-0,723937
44213	26	s	-2,207723	-4,485544	2,580017	4,93686
44214	26	s	1,130696	-2,280546	2,292445	2,551556
44216	26	s	-0,484197	-2,580441	6,842651	7,031941
44217	26	s	-1,715948	-5,4243	6,243169	8,339424
44218	26	s	1,228537	-2,234421	3,820015	4,050771
44219	26	s	-2,563836	-4,842195	4,024932	4,22744
44221	26	s	-7,526148	-18,994717	4,108379	5,307374
44222	26	s	0,60325	-5,634862	5,514228	6,750384
44223	26	s	0,04811	-2,280069	3,846772	4,652691
44224	26	s	1,627855	-4,049724	2,37933	3,33235
44225	26	s	0,242699	-6,07695	2,82655	3,613511
44227	26	s	-1,341754	-9,435589	3,85061	5,740497
44229	26	s	-1,506371	-6,991233	5,422	5,573852
44231	26	s	-0,130411	-10,934361	6,164363	6,057903

Cz	Pz-
1,354921	0,546196
9,361903	6,723257
5,798229	3,631181
0,373948	0,623073
6,606657	9,589032
7,295677	9,270326
11,858151	7,727852
4,18152	3,471898
11,657727	11,95411
0,85104	0,808953
4,836959	1,870533
2,640835	5,006106
9,623903	8,287649
5,551874	4,966349
8,645012	5,815118
8,338947	6,523221
6,973139	8,058175
10,562012	12,528563
5,816063	3,807037
3,281926	3,818916
7,671413	2,70852
6,947233	7,581318
17,464527	13,243537
7,407068	7,087396
1,320746	1,869257
7,510804	-0,354148
10,786203	9,843989
3,567054	3,71904
6,710893	6,385762
18,448624	16,971624
11,066881	10,667064
9,129302	10,508731
5,092081	4,690124
13,591311	8,640839
1,839334	0,845593
7,725543	6,339266
8,474972	6,452648
6,347646	11,137962
3,774762	2,843895
3,841311	3,075312
7,469947	8,267219
8,039428	8,340677
7,671718	4,983999

4,040566	2,908611
6,243697	3,819555
4,214021	1,050086
10,018737	7,375847
7,688149	10,465573
7,143205	9,750152
12,212727	7,929662
11,580618	9,83957
6,381643	1,7595
5,495759	7,26515
1,955231	-1,699339
0,732257	3,047202
8,48368	13,864148
3,164917	2,477982
4,288377	3,389833
3,241161	5,217993
7,75607	8,072977
14,191035	10,388834
8,607146	5,132092
4,914915	3,385149
5,441565	5,58602
2,599161	2,018878
5,648129	3,359971
1,913325	4,283148
7,883213	7,969892
0,530895	5,387594
8,654201	6,930878
2,943981	3,623705
6,156993	2,653194
10,030919	7,973267
4,689578	5,17943
4,416391	3,942058
5,718034	7,349412
5,797775	4,249848
6,98181	6,061924
5,769639	4,612497
4,371147	5,780823
9,269443	10,859037
5,342373	3,456723
4,447793	1,379863
