# Supplementary material for: Violated Expectations in the Cyberball Paradigm: Testing the Expectancy Account of Social Participation With ERP
Source: Front Psychol. 2018 Sep 25;9:1762. doi: 10.3389/fpsyg.2018.01762 (PMC6167485; doi:10.3389/fpsyg.2018.01762)
Supplement: Supplementary file 7 [file Data_Sheet_7.pdf]

Data Sheet 7
ERP effect of targets in the upper and lower visual field in a passive oddball task

Control for the effect of verticality: Passive oddball task

Block I: Target (B) superior

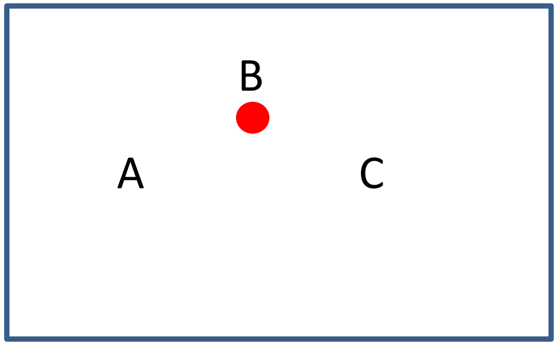

Setup of a passive oddball task

Red dot appears at position A (p=.42), C (p=.42), or B (p=.16)

Task: Count event B

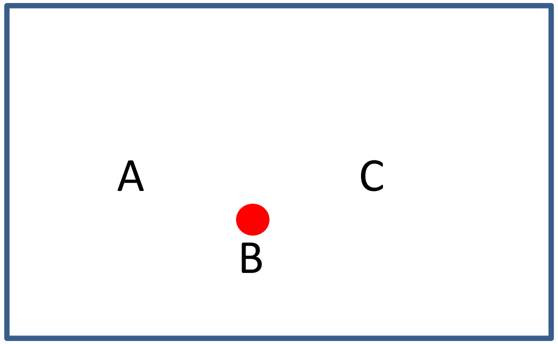

Block II: Target (B) inferior

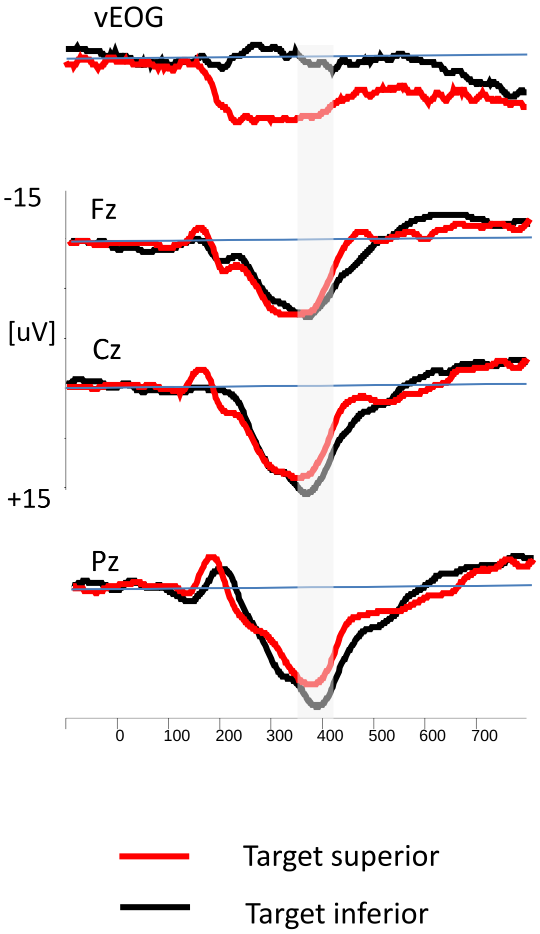

Pilot study (n=14)
Repeated-measure design
Counterbalanced order

Analysis 340 – 420 ms

vEOG

Superior: M = -6.18 uV, SEM = 1.59

Inferior: M = 0.097 uV, SEM = 1.804

$F(1,13) = 4.944$, $p = 0.045$, $\eta_p^2 = 0.276$

Cz/Pz

Superior: M = 7.72 uV, SEM = 1.18

Inferior: M = 10.09 uV, SEM = 1.19

$F(1,13) = 2.787$, $p = 0.119$, $\eta_p^2 = 0.177$

The verticality effect is not expressed in a passive oddball task with targets in the upper or lower visual field
